# Unravelling the correlation between metal induced aggregation and cellular uptake/subcellular localization of Znsalen: an overlooked rule for design of luminescent metal probes†
†Electronic supplementary information (ESI) available. See DOI: 10.1039/c4sc03824j



**DOI:** 10.1039/c4sc03824j

**Published:** 2015-01-21

**Authors:** Juan Tang, Yuan-Bo Cai, Jing Jing, Jun-Long Zhang

**Affiliations:** a Beijing National Laboratory for Molecular Sciences , State Key Laboratory of Rare Earth Materials Chemistry and Applications , College of Chemistry and Molecular Engineering , Peking University , Beijing 100871 , P. R. China . Email: zhangjunlong@pku.edu.cn ; Fax: +86-10-62767034; b School of Chemistry , Beijing Institute of Technology , Beijing 100081 , P. R. China

## Abstract

Unravelling the unique effects of metal coordination on biological behaviours is of importance to design metal based therapeutic and diagnostic agents. In this work, we chose luminescent Znsalen (**ZnL_1_**) as a case study to demonstrate that metal induced aggregation arising from the intermolecular Zn···O interaction influences its cellular uptake and subcellular localization. Comparative studies with the free bases (**L_1_** and **L_2_**) show that **ZnL_1_** undergoes cellular uptake through caveolae-mediated endocytosis and internalizes in endosomal/lysosomal compartments, in contrast to the localization of **L_1_** and **L_2_** in the mitochondria. Further studies of photophysical properties, TEM imaging and DLS analysis suggest that **ZnL_1_** tends to form large sized fibrous structures in aqueous media. To investigate the relationship between **ZnL_1_** aggregation and the biological behaviour, we used pyridine to tune the “aggregation-to-deaggregation” transition and found that, in the presence of pyridine, **ZnL_1_** could localize in the mitochondria and internalize into cells through the passive diffusion pathway. Such distinctive biological behaviours resulting from the different Znsalen species clearly point out the importance of metal induced aggregation or metal speciation analysis in designing metal complexes as biological probes.

## Abstract 2

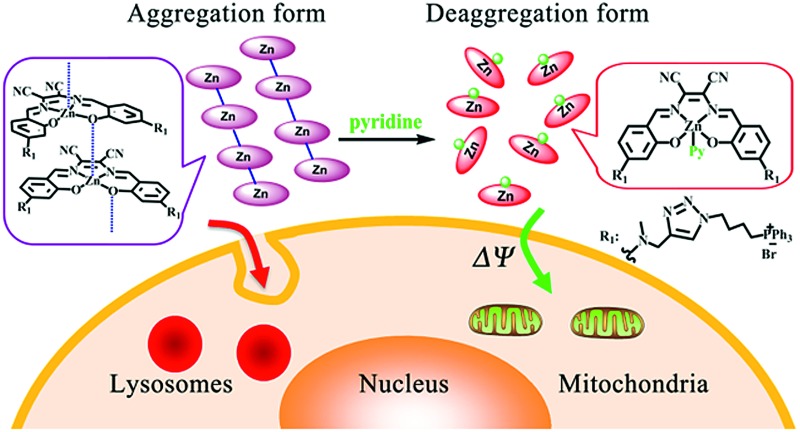
We demonstrate the importance of speciation of luminescent metal complexes in water on biological behaviours such as cellular uptake and subcellular localization.

## Introduction

Luminescent metal complexes have emerged as an important class of imaging agents, because of their photophysical properties such as high luminescence, good photostability, large Stokes shifts and long life-times.^1–10^ Moreover, metal coordination gives luminescent metal complexes greater flexibility and diversity in structures, compared to organic fluorophores.^6,7,11–13^ Thus, deciphering the structural rules governing their biological behaviours is of importance to explore the features of luminescent metal complexes in molecular imaging. Despite the tremendous progress which has been made in understanding the relationships between structural information arising from coordination, such as lipophilicity, oxidation state, charge state and coordination mode, and biological behaviours, much less attention has been given to the physical state or association of luminescent metal complexes in aqueous media.^3,7,9,14^ For metal complexes, the intermolecular metal–ligand or metal–metal interactions reinforce the aggregation process, which may influence the mobility of the metal complex, and therefore constrain the pathways of exposure to the cell and affect the subcellular distribution. Thus, the chemical and physical form of a metal complex in aqueous solution might be an important factor in designing luminescent imaging agents. To address this issue, we herein chose the Znsalen complex (salen = *N*,*N*′-bis(salicylidene)ethylenediamine) as a case study to demonstrate that metal induced aggregation arising from intermolecular Zn···O interaction indeed influences its cellular uptake and subcellular localization.

Znsalens were selected because Zn^2+^ ions possess high Lewis acidity within a planar geometry that allows for extra coordination of a Lewis base ligand or coordinating solvent, or in their absence, self-assembly through an intermolecular Zn···O axial coordination to the phenolic group of another unit.^15–22^ Such speciation of Znsalens or salophens is accompanied by changes in morphology and “switched off/on” fluorescence corresponding to the “aggregation-to-deaggregation” transition.^15,21,23–25^ This forms a chemical basis to design optical sensors to detect anions, biological alkaloids, and even metal cations.^23,26–30^ Extending this prominent feature to living cell imaging, Znsalens could be used as low cytotoxicity agents, and display high fluorescence intensity in specific organelles, in which the environment is more hydrophobic than in cytosol or cell culture media.^31–34^ Since Znsalen tends to aggregate in aqueous media, it is feasible to investigate whether and how Znsalen aggregation affects its biological behaviours such as cellular uptake and subcellular location. This would be important to further design luminescent Znsalen complexes as bioprobes,^35,36^ combined with the insights gained from the coordination chemistry of Znsalens.

**Scheme 1:**
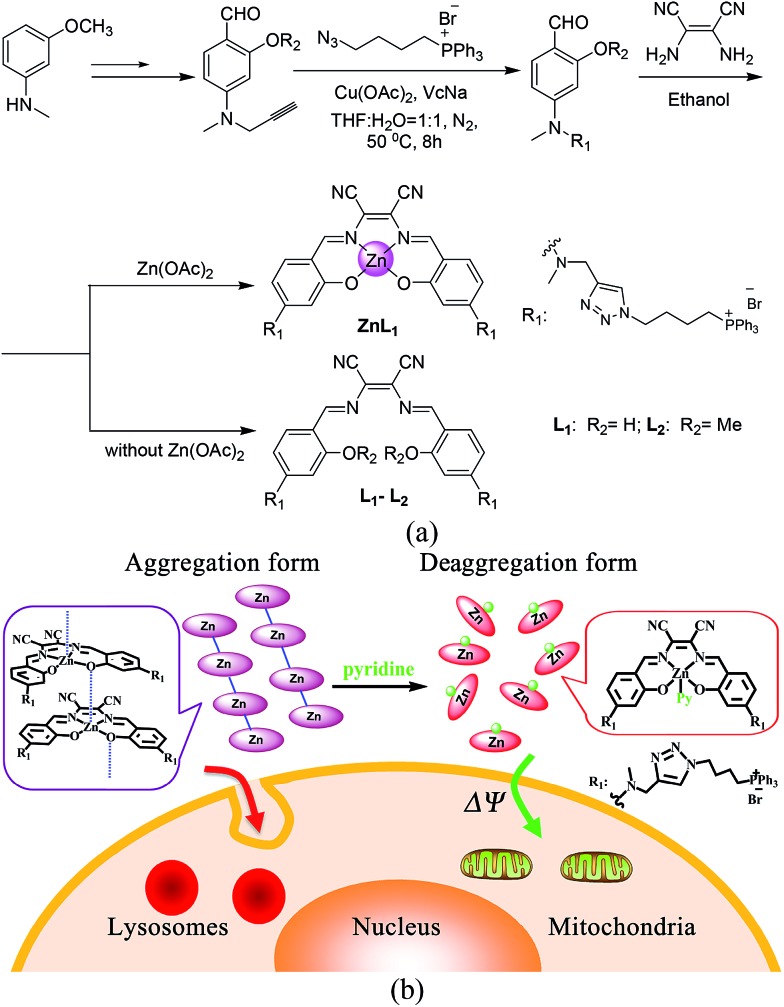
(a) Synthetic routes and chemical structures of **ZnL_1_**, **L_1_** and **L_2_**. (b) Aggregation and deaggregation forms of **ZnL_1_** in aqueous media lead to different cellular uptake pathways and subcellular localization.

To demonstrate the effect of Zn coordination on biological behaviours, we performed comparative studies of cell imaging experiments between a water-soluble Znsalen, **ZnL_1_**, which is conjugated with the mitochondria-targeting triphenylphosphonium cation (TPP), and its free base congeners **L_1_** and **L_2_** (Scheme 1a).^37–42^ Distinctive subcellular distribution between **ZnL_1_** (lysosomal/endosomal compartments) and the free bases **L_1_** and **L_2_** (mitochondria) indicated the significant biological effect arising from Zn coordination. Using **L_1_** and **L_2_** as controls, we excluded the effects of factors such as lipophilicity and charge state on biological behaviours. Then, we hypothesized that the intermolecular Zn···O interaction driving **ZnL_1_** aggregation plays an important role in its distinctive biological behaviours, according to the photophysical properties and morphology of **ZnL_1_** and **L_1_** in aqueous media. To confirm this hypothesis, we used pyridine as an extra ligand to dissociate the aggregates by breaking the intermolecular Zn···O interaction, and found that **ZnL_1_** undergoes the passive diffusion pathway and mainly localizes in mitochondria (Scheme 1b). Thus, these results point out the importance of metal induced aggregation in determining biological behaviours, which is potentially useful to the design of luminescent metal probes with organelle specificity.

## Results and discussion

**ZnL_1_** and **L_1_** were synthesized according to Scheme 1a. N-substituted salicylaldehyde was obtained from 3-methoxyl-*N*-methylaniline and propargyl bromide, followed by Vilsmeier–Haack formylation. TPP was introduced using the copper-catalyzed “click” reaction. **ZnL_1_** was synthesized through a “one pot” reaction of salicylaldehyde, diimine and zinc acetate, while **L_1_** was obtained in the absence of zinc acetate. To increase the lipophilicity of **L_1_**, we prepared **L_2_** as a control using salicylaldehyde with a methyl-group-protected phenol moiety (ESI†). The detailed synthetic procedure and characterization using ^1^H NMR, ^13^C NMR, ESI-MS, UV-vis and IR are given in the ESI.†

**Fig. 1:**
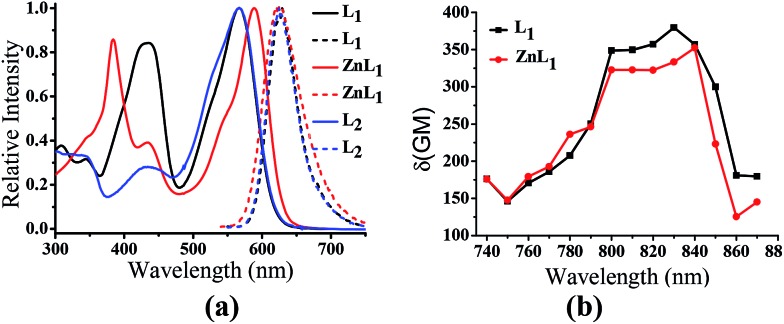
(a) Normalized UV-vis and fluorescence spectra of **ZnL_1_**, **L_1_** and **L_2_** in DMSO. *λ*_ex_ = 380 nm. (b) Two photon induced absorption cross-section of **ZnL_1_** and **L_1_** in DMSO using Rhodamine B as a reference.

As shown in Fig. 1a, the absorption spectra of **ZnL_1_**, **L_1_** and **L_2_** in DMSO solution (2 × 10^–5^ M) show two major absorption bands between 350–450 and 500–600 nm. The free bases **L_1_** and **L_2_** display broad absorption bands from 360 to 470 nm, whereas **ZnL_1_** shows a sharp band centered at 385 nm with a low-energy shoulder at 435 nm. The low-energy bands are likely due to an internal charge transfer (ICT) transition. **ZnL_1_**, **L_1_** and **L_2_** exhibit red emission (*λ*_max_ = 623, 631 and 624 nm for **ZnL_1_**, **L_1_** and **L_2_** in DMSO, respectively) with fluorescence quantum yields of 0.26, 0.29 and 0.21 (Table S1†). The ^1^H NMR spectra of **ZnL_1_**, **L_1_** and **L_2_** in *d* ^6^-DMSO display sharp signals with the expected multiplicities for their molecular structures (Fig. S15–S17†). Following Di Bella's procedure,^21^ we used diffusion-ordered spectroscopy (DOSY) NMR to estimate the molecular weight of **ZnL_1_** in *d* ^6^-DMSO (Table S4 and Fig. S18†). The obtained molecular weight is *ca.* 1260, close to the calculated molecular weight. These results clearly suggest that monomeric **ZnL_1_** is the main species in the coordinating solvent DMSO. In addition, the lipophilicities of **ZnL_1_**, **L_1_** and **L_2_** were measured using the logarithm of their octanol/water partition coefficients (log *Ps* 0.12, –0.77, 0.09) according to Leo's method.^43^ This suggests that Zn coordination increases the lipophilicity of **L_1_**, by an amount comparable to the methoxylation of the phenolic group of **L_1_** (Table S2†).

### Zn coordination affects cellular uptake and subcellular distribution

**Fig. 2:**
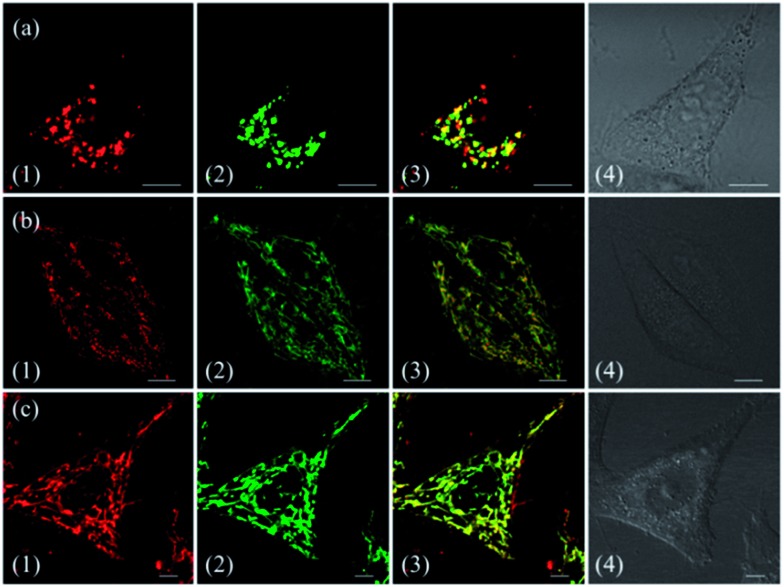
Co-localization analysis of (a) **ZnL_1_** with lysosome tracker and (b and c) **L_1_** or **L_2_** with mitochondria tracker. (1) Fluorescence images of **ZnL_1_**, **L_1_** and **L_2_**, ex: 543 nm; (2) fluorescence images of commercial trackers LysoTracker® Green DND-26 in (a) or MitoTracker Green in (b and c), ex: 488 nm; (3) merged images of (1) and (2); (4) differential interference contrast channel. Scale bar: 10 μm.

To demonstrate the effect of Zn coordination on biological behaviours, we investigated the intracellular distribution of **ZnL_1_**, **L_1_** and **L_2_** in HeLa cells using confocal laser scanning microscopy (CLSM). FYVE-EGFP, EHD1-EGFP, LysoTracker® Green DND-26 and MitoTracker Green served as markers for the early endosome, late endosome, lysosome and mitochondria organelles, respectively. As shown in Fig. 2a and S1–3,† **ZnL_1_** showed good cell permeability and was mainly distributed in the lysosomal/endosomal compartments. It exhibited a co-localization level of approximately 0.56 with LysoTracker® Green DND-26. Interestingly, **L_1_** and **L_2_** particularly stained mitochondria with Pearson's correlation coefficients of 0.92 and 0.83 (Fig. 2b and c). The similar subcellular distributions of **L_1_** and **L_2_** clearly suggested that the lipophilicity of the salen ligands is not the main factor that affects their subcellular distribution.

**Fig. 3:**
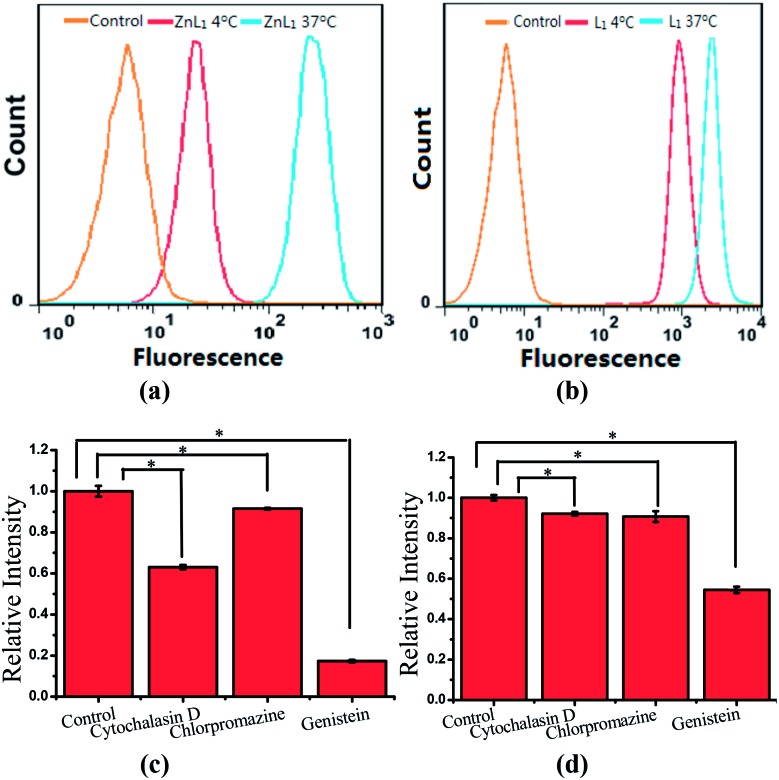
Cellular uptake of **ZnL_1_** and **L_1_**, analysed using flow cytometry. Internalization of **ZnL_1_** (a) or **L_1_** (b) was investigated at 4 or 37 °C. HeLa cells were treated with cytochalasin D (5 μg mL^–1^), chlorpromazine (10 μg mL^–1^) or genistein (100 μM) for 30 min and then incubated with inhibitor and **ZnL_1_** or **L_1_** (2 μM) for 1 h. Cells treated with **ZnL_1_** or **L_1_** only were used as controls. Mean relative intracellular fluorescence intensities of the intracellular uptake of **ZnL_1_** (c) and **L_1_** (d) are shown as histograms (*n* = 3, *P < 0.001).

To explore the effect of Zn coordination on the cellular uptake pathway, the temperature-dependent cellular uptake of **ZnL_1_** or **L_1_** in HeLa cells was carried out at 4 or 37 °C, and analysed using flow cytometry. As shown in Fig. 3a and b, the cellular internalization of **ZnL_1_** at 4 °C showed a significantly lower intracellular level compared to that of the cells incubated in parallel at 37 °C, whereas **L_1_** was less affected by temperature. This indicates that the cellular internalization of **ZnL_1_** is an active process despite the low intracellular luminescence in cells incubated at 4 °C. In contrast, passive diffusion might be the dominant mechanism for the free base **L_1_**, although a background level of active transport also occurs.

**Fig. 4:**
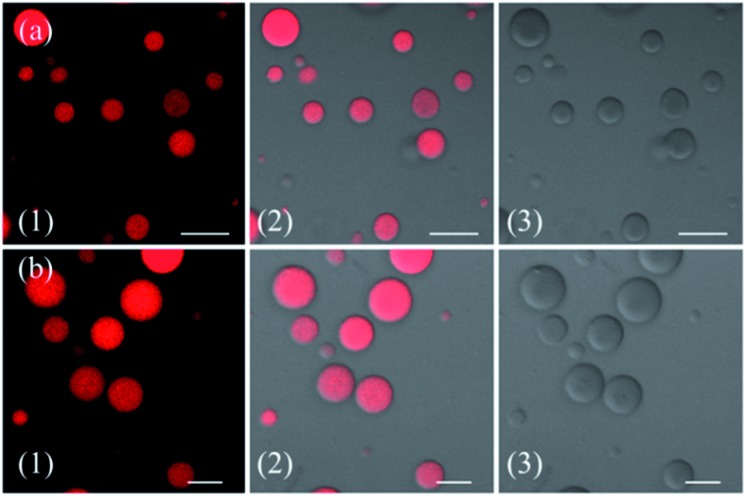
Mitochondria permeability investigation of **ZnL_1_** and **L_1_** with GUVs as mitochondria membrane mimics. Confocal images of (a) **ZnL_1_** and (b) **L_1_** confined in GUVs. (1) Fluorescence images of **ZnL_1_** and **L_1_**; (2) merged images of (1) and (3); and (3) differential interference contrast channel. Scale bar: 10 μm.

To demonstrate the cellular internalization of **ZnL_1_**, we then used flow cytometry, applying the endocytosis inhibitors chlorpromazine (inhibitor of clathrin-mediated endocytosis),^44–46^ genistein (inhibitor of caveolae-mediated endocytosis),^44,47,48^ or cytochalasin D (inhibitor of macropinocytosis).^49–53^ As Fig. 3c and d show, the treatment with 100 μg mL^–1^ genistein resulted in a drastic decrease of 80% in the cellular uptake of **ZnL_1_**, suggesting that **ZnL_1_** is mainly internalized *via* caveolae-mediated endocytosis, which plays a general role in the internalization of large sized particles into cells.^52,54,55^ In addition, treatment with cytochalasin D also led to a 40% decrease in cellular uptake, suggesting that a macropinocytosis-mediated pathway might also be partly involved (Fig. 3c). In contrast, for **L_1_**, treatment with genistein only resulted in a 45% decrease in the cellular uptake, and no obvious effect was observed when treated with cytochalasin D or chlorpromazine, which indicates that **L_1_** partly undergoes caveolae-mediated endocytosis and may mainly cross the cell membrane by passive transport, and then internalize in the mitochondria (Fig. 3d). Furthermore, to exclude the possibility that zinc coordination diminishes the mitochondrial permeation capability, we examined the ability of **ZnL_1_** and **L_1_** to stain isolated mitochondria. Following Schagger's procedure,^56^ we isolated mitochondrial fractions and confirmed the activity using JC-1, shown in Fig. S4.† After the incubation of **ZnL_1_** and **L_1_** with active mitochondria for 0.5 h, the fluorescence of the resuspended mitochondria was detected (Fig. S5†), indicating the mitochondrial permeation capability of **ZnL_1_** and **L_1_**. To further verify this, we also used giant unilamellar phospholipid vesicles (GUVs) as mitochondria models following Pielak's method (Fig. S6†).^57^ As shown in Fig. 4, both **ZnL_1_** and **L_1_** permeated the mitochondria mimic membranes and localized in the GUV matrix after 20 min. These results suggested that Zn coordination does not affect the mitochondria permeation capability, and the different subcellular distributions of **ZnL_1_** and **L_1_** might be due to their cellular uptake pathways.

### Photophysical properties and morphology in aqueous media

**Fig. 5:**
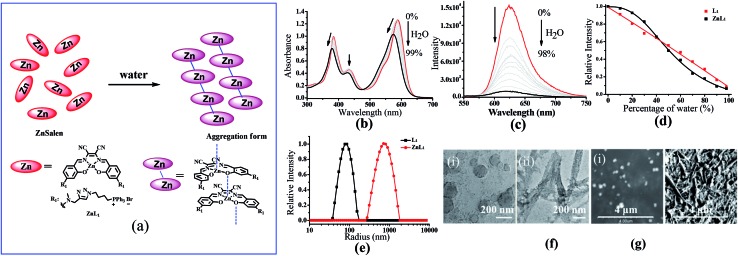
(a) The change of speciation of **ZnL_1_** in water solution. (b) UV-vis and (c) fluorescence spectra (*λ*_ex_ = 380 nm) of **ZnL_1_** (20 μM) in mixed solutions of H_2_O/DMSO. The content of H_2_O was varied from 0% to 99%. (d) Fluorescence changes of **ZnL_1_** and **L_1_** with different water percentage. (e) DLS analysis of **ZnL_1_** and **L_1_** in water containing 0.1% DMSO. (f) TEM images of self-assembled (i) **L_1_** and (ii) **ZnL_1_** (scale bar: 200 nm). (g) SEM pictures of two assemblies of (i) **L_1_** and (ii) **ZnL_1_** (scale bar: 4 μm).

Since cellular uptake of **ZnL_1_** is related to caveolae-mediated endocytosis and macropinocytosis, we envisioned that the behaviour of **ZnL_1_** in aqueous media might influence the cellular uptake and subcellular distribution. To understand this behaviour, we carried out UV-vis absorption and emission spectroscopy of **ZnL_1_** and **L_1_** (Fig. 5b and c, S7 and S8†) in aqueous media. With increasing water content in the DMSO solution, **ZnL_1_** and **L_1_** displayed blue shifts of 15 nm and 26 nm in the low-energy absorption spectrum, and the emission was quenched gradually, with blue shifts of 6 and 11 nm, respectively. The lack of a clear isosbestic point in the absorption spectra and the quenched fluorescence for **ZnL_1_** and **L_1_** suggested the formation of aggregates in aqueous media.^19,58–60^ Interestingly, plotting the emission intensities of **ZnL_1_** and **L_1_** *versus* the water contents (%) showed a logistic regression with *P* values of 2.71 and 1.04 (Fig. 5d), respectively. The larger *P* value for **ZnL_1_** indicated a cooperative effect on the formation of **ZnL_1_** aggregates in aqueous media.

The ^1^H NMR spectra of **ZnL_1_** and **L_1_** (concentration: 1 mM) in the mixtures of D_2_O and *d* ^6^-DMSO solvents (Fig. S11†) show that the signals of the protons of the salen skeleton became broadened and their intensity decreased progressively with increasing D_2_O content, which is probably due to restricted motion of the hydrophobic salen moiety, whereas the signals of the triphenylphosphonium group did not significantly change. In addition, diffusion-ordered spectroscopy (DOSY) NMR for **ZnL_1_** in *d* ^6^-DMSO solution containing 30% D_2_O showed an average molecular mass of 1602 (Table S4 and Fig. S19†), indicating partial aggregation of **ZnL_1_** under these conditions. However, for the broad and weak signals, we cannot obtain reliable DOSY data at higher concentrations of D_2_O, which are predicted to form larger aggregates. Nevertheless, optical spectroscopy data and NMR spectroscopic studies clearly indicate the “deaggregation to aggregation” transition of **ZnL_1_** when the solvent was switched from DMSO to water.

To further examine the morphology of **ZnL_1_** or **L_1_** in aqueous media, dynamic light scattering (DLS) experiments were performed. As shown in Fig. 5e, **L_1_** (1 μM) has a smaller hydrodynamic diameter (*D*_r_, *ca.* 60 nm) and narrower distribution than **ZnL_1_** (*D*_r_, *ca.* 720 nm). No change of the **ZnL_1_** or **L_1_** aggregates were observed in cell culture media (Fig. S9†), indicating that the ingredients in the cell culture media have little effect on the aggregates. Then, the morphologies of **ZnL_1_** and **L_1_** aggregates were studied by transmission electron microscopy (TEM) and scanning electron microscopy (SEM) by drop-casting the aqueous solutions of **ZnL_1_** or **L_1_** onto Cu–C (200) substrates. As shown in Fig. 5f and g, the TEM and SEM images clearly show the formation of a fibrous nanostructure (with dimensions of 43.5 ± 2.6 nm and more than 1 μm in length) for **ZnL_1_**, while **L_1_** shows a distinct globular morphology with dimensions ranging from 20 nm to 70 nm, which are consistent with the DLS results. To exclude the effect of lipophilicity, we used **L_2_** as a control, and **L_2_** exhibited a similar particle size and distribution to those of **L_1_** in both aqueous solution and cell culture medium (Fig. S9 and 10†). Given the similar structures of **ZnL_1_**, **L_1_** and **L_2_**, we ascribed the different morphologies in aqueous media to the intermolecular Zn···O interaction in **ZnL_1_** aggregates (Scheme 1b).

Although the aggregation of Znsalen complexes through the intermolecular Zn···O interaction in non-coordinating solvents is a usual feature, such studies in water have much less been reported. There are several examples of water soluble Znsalens that were found to be monomeric species in aqueous solutions,^27,28,61^ and thus the effect of water on the aggregation of **ZnL_1_** in water needs to be further discussed. Generally, water is a Lewis base and a coordinating solvent, and can compete with the intermolecular Zn···O interaction, and hence may prevent aggregation. However, another facet of water as solvent, in which the hydrophobic effect of Znsalen promotes such aggregation, cannot be neglected. Obviously, the role of water is complicated and highly dependent on the different binding affinities of the H_2_O–Zn and the intermolecular phenolic O–Zn interaction, the structure and lipophilicity of Znsalens, the properties of the conjugates and the solvent effect. In this work, the formation of **ZnL_1_** aggregates in water, confirmed by UV-vis absorption, fluorescence and NMR spectroscopic studies, together with morphological studies clearly indicates that the intermolecular Zn···O interaction is more prominent than the coordination of water. On the other hand, since lipophilic triphenylphosphonium cations were introduced, **ZnL_1_** is to some extent hydrophobic (log *P* 0.12). The flexible spacer (C4 chain and triazole linker) promotes the aggregation of the Znsalen moiety and the triphenylphosphonium cations as head groups located in the interfaces, which is in agreement with the changes of the proton signals in the ^1^H-NMR spectra. Thus, the hydrophobic effect is another important factor that determines **ZnL_1_** aggregation in water.

### Stability of **ZnL_1_** aggregates in aqueous media

**Fig. 6:**
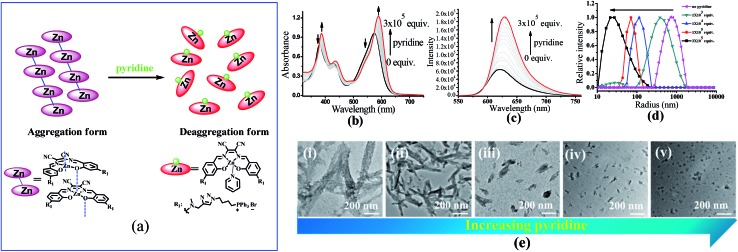
(a) The change of speciation of **ZnL_1_** in the presence of pyridine. The dissociation of the aggregated state of 20 μM **ZnL_1_** induced by competitive pyridine binding monitored using (b) UV-vis and (c) fluorescence spectra. (d) DLS analysis of **ZnL_1_** (1 μM) in water with the addition of 0–300 thousand equiv. pyridine. (e) TEM images of self-assembled **ZnL_1_** in the presence of (i–v) 0, 1 × 10^3^, 1 × 10^4^, 1 × 10^5^, 3 × 10^5^ equiv. pyridine. Scale bar is 200 nm.

To understand the contribution of the intermolecular Zn···O interaction to **ZnL_1_** aggregation, we carried out competitive pyridine binding experiments. According to previous studies,^15,17–19^ the competitive binding of Lewis bases such as pyridine would dissociate the aggregates if the intermolecular Zn···O interaction was the main driving force for the self-assembly process in non-coordinating solvents. As shown in Fig. 6b, when pyridine was added to an aqueous solution of **ZnL_1_**, the bands at around 380 and 550 nm were red shifted to 387 and 590 nm, indicating the dissociation of the **ZnL_1_** aggregates. As expected, the fluorescence intensity increases along with the increasing amount of pyridine (Fig. 6c). Fluorescence lifetimes were recorded before and after pyridine addition. In water, the luminescence decays are fitted as double-exponential decays with lifetimes of 2.0 (76%) and 5.2 (24%) ns. After pyridine titration, the fluorescence lifetime is 4.0 ns fitted as a single-exponential decay, slightly shorter than that in DMSO (5.2 ns). The complete deaggregation of **ZnL_1_** requires up to 2 × 10^5^ equiv. pyridine (Fig. S12†), which is comparable to the extremely stable bimetallic Znsalen complex reported by Kleij and coworkers.^20^ Monitoring the pyridine titration using ^1^H NMR spectra demonstrated the sharpened proton signals of **ZnL_1_** with downfield shifts (Fig. S13†), also supporting the deaggregation of **ZnL_1_** by pyridine. Moreover, the dissociation of **ZnL_1_** aggregates is evident from DLS results and TEM imaging. Fig. 6d shows the decrease in hydrodynamic radius from 720 nm to 27 nm with increasing pyridine from 0 to 300 thousand equiv. TEM imaging confirmed this trend and showed that the size of aggregates was reduced from 1250 ± 117 to 31 ± 2.3 nm (Fig. 6e).

**Fig. 7:**
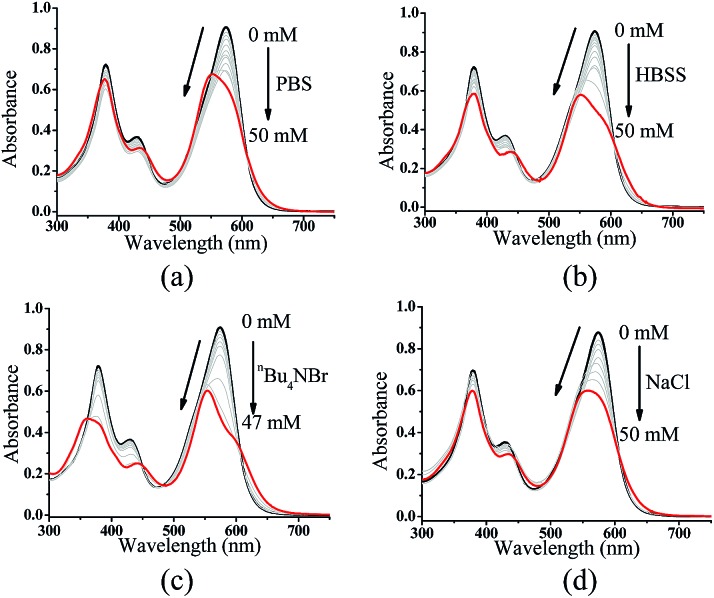
UV-vis spectra of 20 μM **ZnL_1_** in the presence of (a) 0–50 mM PBS, (b) HBSS, (c) ^*n*^Bu_4_NBr, or (d) NaCl.

In addition to pyridine, we attempted to test the effect of anions on **ZnL_1_** aggregation in aqueous media. Previous studies demonstrated that anions such as phosphate^23,27,61^ and Br^–^^25^ are able to coordinate Zn and dissociate Znsalen aggregates, however, with much smaller binding constants than pyridine.^61,62^ In this work, we chose PO_4_^3–^ (PBS buffer), Br^–^ (^*n*^Bu_4_NBr) and HBSS buffer (containing various inorganic anions), which are relevant to biological studies. As shown in Fig. 7a–c, with increasing the concentration of PBS, ^*n*^Bu_4_NBr and HBSS from 0 to 50 mM, the UV-vis spectra showed increasing bandwidths and blue-shifted with decreasing intensity at 500–600 nm, indicating increased aggregate formation. To understand this, we proposed that the high concentration of anions promoting the **ZnL_1_** aggregation is due to a salt effect arising from PBS, ^*n*^Bu_4_NBr and HBSS. To test this, we measured the UV-vis spectra of **ZnL_1_** in the presence of NaCl (0–49 mM). As shown in Fig. 7d, similar spectroscopic changes were observed. Thus, we concluded that the aggregation of **ZnL_1_** might be affected by the ionic strength of these salts. This also could be used to explain why pyridine is very effective to disassociate **ZnL_1_** for the high binding constant to Zn ion and negligible ionic strength of pyridine. Although the “aggregation-to-deaggregation” process for Znsalen/salophens has been extensively studied,^15,18–21^ few studies on the stability of such aggregates in aqueous media have been performed. This is important to verify whether the aggregates are stable enough to affect the biological behaviours. Following Kleij's method,^18,20^ we also estimated the stability constants of the **ZnL_1_** aggregates in aqueous media. The relationship of the overall aggregation constant *K*_agg_, stepwise aggregation constant *K*_*n*/*n*+1_, and the aggregation number *n* can be described using the following equation:

1 (Znsalen)_*n*_ + *n*Py ⇌ *n*Znsalen(Py) *K* = *K*_Py_^*n*^/*K*_agg_, *K*_agg_ = *K*_*n*/*n*+1_^*n*–1^

In this equation, *K*_py_ represents the coordination constant of pyridine to the Zn center of **ZnL_1_**. To evaluate *K*_py_, we carried out pyridine titration on the solution of **ZnL_1_** in dichloromethane (DCM) (Fig. S14†). The coordination constant was calculated to be 1.4 × 10^4^ M^–1^, which is lower than those (5.3 and 5.9 × 10^5^) reported by Kleij,^18,26^ and the value of 4.7 × 10^6^ reported by Di Bella.^62^ The lower coordination constant was probably due to the interference of the large triphenylphosphonium groups. As in the pyridine titration in aqueous solution, the increase at 590 nm was monitored to calculate the total reaction constant *K*. *K*_agg_ and *K*_*n*/*n*+1_ depend on the aggregation number *n*. Table S3† lists all the calculated values of *K*_agg_ and *K*_*n*/*n*+1_ with increasing aggregation numbers. Each addition of monomer to a previously formed oligomer has a high association constant, in the order of about 10^10^ M^–1^, which is about 2 orders higher than for the dimerization of Znsalens in organic solvents.^18^ This clearly demonstrates that the solvent effect reinforces the intermolecular Zn···O interaction and enhances the stability of the **ZnL_1_** aggregates.

### Effect of the “aggregation/deaggregation” process on the biological behaviours

**Fig. 8:**
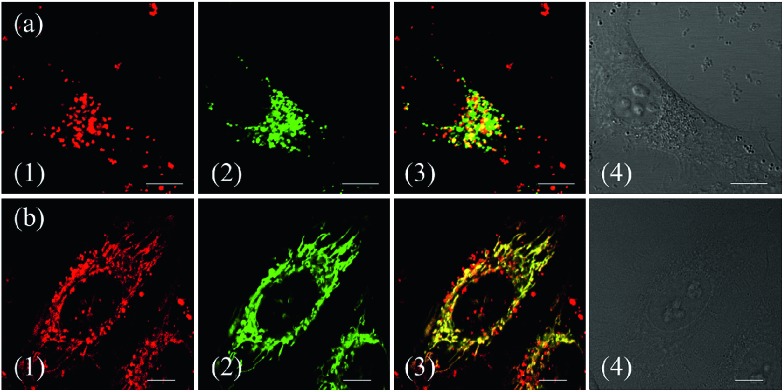
Co-localization analysis of (a) **ZnL_1_** in the absence of pyridine with lysosomes and (b) **ZnL_1_** in the presence of pyridine with mitochondria: (1) fluorescence images of **ZnL_1_**, ex = 543 nm; (2) fluorescence of commercial trackers LysoTracker® Green DND-26 in (a) or MitoTracker Green in (b), ex = 488 nm; (3) merged picture of (1) and (2); (4) differential interference contrast channel. Scale bar: 10 μm.

To confirm the hypothesis that **ZnL_1_** aggregation affected its biological behaviours, we performed cell imaging experiments using **ZnL_1_** in the presence or absence of pyridine. According to the above results, **ZnL_1_** aggregates can be dissociated by the addition of pyridine, which would exhibit different cellular uptake pathways and subcellular distributions from the sole use of **ZnL_1_**. The intracellular luminescence and subcellular distribution of **ZnL_1_** were investigated in HeLa cells using confocal laser scanning microscopy (CLSM). As shown in Fig. 8a, in the absence of pyridine, **ZnL_1_** exhibited punctate red fluorescence in the lysosomal/endosomal compartments, which were mainly co-localized with LysoTracker® Green DND-26 (Pearson's correlation coefficient 0.56). In contrast, in the presence of pyridine (about 6000 equiv.), **ZnL_1_** was mainly localized in the mitochondria and co-localized with commercial MitoTracker Green (Pearson's correlation coefficient 0.60) (Fig. 8b). These results clearly demonstrate that the “aggregation/deaggregation” transition influences the subcellular distribution.

**Fig. 9:**
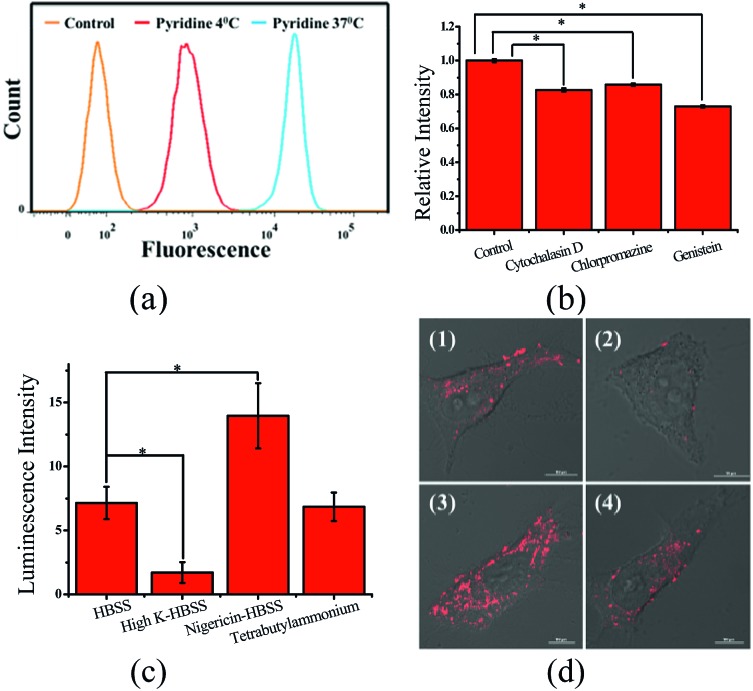
Cellular uptake of **ZnL_1_** analysed by flow cytometry and CLSM. (a) Effect of incubation temperature on the internalization of **ZnL_1_** in the presence of pyridine was investigated at 4 or 37 °C. (b) Effect of endocytosis on the internalization of **ZnL_1_** in the presence of pyridine: HeLa cells were treated with cytochalasin D (5 μg mL^–1^), chlorpromazine (10 μg mL^–1^) or genistein (100 μM) for 30 min and then incubated with inhibitor and **ZnL_1_** (2 μM) for 1 h. Cells treated with **ZnL_1_** only were used as controls. (c) and (d) Effect of the plasma membrane potential and organic cation transporter inhibitor on the internalization of **ZnL_1_**: HeLa cells treated with **ZnL_1_** in the presence of pyridine buffered in (1) HBSS, (2) high K^+^-HBSS, (3) 5 μM nigericin-HBSS (cells were treated with 5 μM nigericin first for 30 min) or (4) 1 mM tetrabutylammonium bromide-HBSS (cells were treated with 1 mM tetrabutylammonium bromide first for 20 min). Mean relative intracellular fluorescence intensities of intracellular uptake are shown in histograms (*n* = 3, *P < 0.001). Scale bar: 10 μm.

To understand whether the deaggregation of **ZnL_1_** in aqueous solution influences the internalization mechanism, we examined the temperature-dependent cellular uptake using flow cytometry. As shown in Fig. 9a, a significantly lower intracellular fluorescence intensity was observed for **ZnL_1_** in the presence of pyridine at 4 °C than that at 37 °C, indicating that the **ZnL_1_** species were internalized by cells *via* a temperature-dependent process. Then, we employed the endocytosis inhibitors chlorpromazine (inhibitor of clathrin-mediated endocytosis),^44–46^ genistein (inhibitor of caveolae-mediated endocytosis),^44,47,48^ and cytochalasin D (inhibitor of macropinocytosis)^49–53^ as described above, to investigate whether **ZnL_1_** internalizes into the cell through endocytosis. As shown in Fig. 9b, in the presence of pyridine, the treatments with genistein, cytochalasin D or chlorpromazine only resulted in a moderate decrease of intracellular fluorescence (27%, 17% and 14%, respectively) and thus cellular uptake of **ZnL_1_** in the presence of pyridine is almost not correlated to endocytosis, which is markedly different from the endocytosis pathway of **ZnL_1_** in the aggregated state.

Given that the TPP conjugate possesses a positive charge, the internalization of **ZnL_1_** with pyridine may be facilitated by organic cation transporters (OCT) or driven by the plasma membrane potential (–50 to –70 mV, negative inside).^63–66^ When using tetrabutylammonium bromide as the OCT inhibitor,^67^ HeLa cells showed no obvious intracellular fluorescence change (Fig. 9c and d). To investigate the effect of the plasma membrane potential, we examined the cellular uptake of **ZnL_1_** in the presence of pyridine under the conditions of high potassium buffer (K^+^-HBSS, 170 mM K^+^, depolarization)^65,67,68^ or nigericin-HBSS (10 μM, hyperpolarization).^65,69^ As shown in Fig. 9c and d, the K^+^-HBSS-treated cells displayed a remarkable decrease in intracellular fluorescence (80%), while the nigericin-treated cells showed an intracellular fluorescence increase of nearly 2 fold. This clearly demonstrated that **ZnL_1_** in the presence of pyridine is internalized mostly by membrane potential dependent passive diffusion. Since we have demonstrated that PBS, HBSS, and Br^–^ cannot lead to **ZnL_1_** deaggregation and couldn't disturb pyridine coordination, combined with its photophysical properties and morphology in aqueous media, we hypothesized that the distinctive internalization pathways may be due to the different morphologies between the “aggregation/deaggregation” transition of **ZnL_1_**, because the caveolae-mediated or macropinocytosis-mediated endocytosis is related to the uptake of large sized particles into cells.^44,66,68^

## Conclusions

Taking our results together, we demonstrated that the intermolecular Zn···O interaction between Znsalen played an important role in determining its cellular uptake pathway and subcellular distribution. Through comparative studies between **ZnL_1_** and the free bases **L_1_** and **L_2_**, Zn coordination was found to lead to a distinctive cellular uptake pathway and subcellular distribution. More importantly, the photophysical and morphology studies on **ZnL_1_** and the free bases in aqueous media suggest that the different aggregation states arising from the intermolecular Zn···O interaction play a critical role in influencing the biological behaviours. This hypothesis was confirmed by cell imaging experiments using **ZnL_1_** in the presence or absence of pyridine, which clearly demonstrated the effect of tuning the “aggregation/deaggregation” transition of **ZnL_1_** in aqueous media on the cellular uptake pathway and subcellular distribution. These results point to a new factor, “metal induced aggregation”, which effectively influences cellular uptake and subcellular distribution, and which should not be overlooked in designing luminescent metal complexes as biological probes.
