# Supplementary material for: Unravelling the correlation between metal induced aggregation and cellular uptake/subcellular localization of Znsalen: an overlooked rule for design of luminescent metal probes
Source: Chem Sci. 2015 Jan 21;6(4):2389–97. doi: 10.1039/c4sc03824j (PMC5488197; doi:10.1039/c4sc03824j)
Supplement: Supplementary file 1 [file SC-006-C4SC03824J-s001.pdf]

Electronic Supplementary Material (ESI) for Chemical Science.
This journal is © The Royal Society of Chemistry 2015

Supporting Information

Unravelling the Correlation between Metal Induced Aggregation and Cellular Uptake/Subcellular Localization of Znsalen: An Overlooked Rule for Design of Luminescent Metal Probes

Juan Tang, [a] Yuan-Bo Cai, [a] Jing Jing[b] and Jun-Long Zhang* [a]

[a] Beijing National Laboratory for Molecular Sciences, State Key Laboratory of Rare Earth Materials Chemistry and Applications, College of Chemistry and Molecular Engineering, Peking University, Beijing 100871, P.R. China, Fax: +86-10-62767034, E-mail: zhangjunlong@pku.edu.cn.

[b] School of Chemistry, Beijing Institute of Technology, Beijing 100081, P.R. China.

Content

1. Experimental methods 4
1.1 General information 4
1.2 Synthesis 5
1.3 Quantum yield determination 8
1.4 Two photon absorption 8
1.5 Lop P determination 8
1.6 The Mitochondria Isolation 9
1.7 GUV preparation 9
1.8 Cell culture 9
1.9 Colocalization assay 9
1.10 Cell uptake mechanism assay 10
1.11 Estimation of aggregation constants of ZnL_1 11
1.12 DOSY experiments and estimation of molecular mass of ZnL_1 12
2. Supplementary Tables 13
Table S1. Photophysical properties of ZnL_1, L_1 and L_2. 13
Table S2. Lipophilicity (log $P_{o/w}$) of ZnL_1, L_1 and L_2 13
Table S3. Aggregation constants of ZnL_1. 13
Table S4. Diffusion coefficients (D) and molecular mass of ZnL_1 from DOSY 13
3. Supplementary Figures 14
Figure S1. Fluorescence colocalization images of HeLa cells incubated with ZnL_1 14
Figure S2. Time Tracing of cellular behaviors of ZnL_1 15
Figure S3. Time tracing of cellular behaviors of L_1 16
Figure S4. Mitochondrial potential confirmation using indicator JC-1. 17
Figure S5. Mitochondrial permeability investigation of ZnL_1 and L_1 18
Figure S6. GUV characterization 19
Figure S7. Absorption spectra of L_1 in different contents of H_2O and DMSO 20
Figure S8. Emission of L_1 in different contents of H_2O and DMSO 21
Figure S9. DLS analysis of ZnL_1, L_1 and L_2 in FBS medium 22
Figure S10. DLS analysis of L_2 in water. 23
Figure S11. ^1H NMR of ZnL_1 in DMSO-d^6/D_2O mixed solvents. 24
Figure S12. Plots of absorption and emission changes of ZnL_1 by titrating pyridine 25
Figure S13. ^1H NMR of ZnL_1 in D_2O with different equiv. of pyridine-d^5 26
Figure S14. Spectral changes of ZnL_1 by titrating pyridine in DCM 27
Figure S15. ^1H NMR spectrum of ZnL_1 in DMSO-d^6 28
Figure S16. ^1H NMR spectrum of L_1 in DMSO-d^6 29
Figure S17. ^1H NMR spectrum of L_2 in DMSO-d^6 30
Figure S18. ^1H NMR DOSY spectrum of ZnL_1 in DMSO-d^6/D_2O (9:1) 31
Figure S19. ^1H NMR DOSY spectrum of ZnL_1 in DMSO-d^6/D_2O (7:3) 32
3. IR spectra 33
Figure S20. FT-IR spectrum (KBr pallate) of ZnL_1. 33

Figure S21. FT-IR spectrum (KBr pallate) of L_1. .... **34**
Figure S22. FT-IR spectrum (KBr pallate) of L_2. .... **35**
4.^{1}H NMR and ^{13}C NMR spectra **36**
5. HR ESI-MS spectra **45**
6. References **48**

1. Experimental methods

1.1 General information

All solvents and chemicals for synthesis were purchased from Alfa Aesar and J&K and used as received without further purification, unless otherwise specified. Cellular imaging trackers and endocytosis inhibitors were purchased from Invitrogen (Life Technologies). Mitochondrial Isolation Kit for Cultured Cells is purchased from Cultured Cells BiYunTian company.

The ^{1}H NMR spectroscopic measurements were carried out using a Bruker-400 NMR at 400 MHz with tetramethysilane (TMS) as internal reference. The ^{13}C NMR spectroscopic measurements were carried out using a 100 MHz NMR (Bruker-400, USA). Electrospray ionization (ESI) mass spectra were performed on a Fourier Transform Ion Cyclotron Resonance Mass Spectrometer (Bruker, USA), positive-ion mode. FT-IR spectra were taken on a Nicolet iN10 MX Fourier Transform Infrared Spectrometer. The steady-state absorption spectra were obtained with an Agilent 8453 UV-*vis* spectrophotometer in 1cm path length quartz cells. Single-photon luminescence spectra were recorded using fluorescence lifetime and steady state spectrophotometer (Edinburgh Instrument FLS920). Quantum yields of one photon emission of all the synthesized compounds were measured relative to the fluorescence of Rhodamine B (Φ=0.65) in ethanol, and the two photon absorption cross section of the probes was calculated at each wavelength relative to Rhodamine B as standard. Confocal fluorescent images of living cells were performed using Nikon A1R-si Laser Scanning Confocal Microscope (Japan), equipped with lasers of 405/488/543/638 nm. Morphology images of ZnL_1 and L_1 were taken using Transmission Electron Microscope (JEM-2100, Japan) and Scanning electron microscope (SPI3800/SPA400, Japan). Zeta plus were used to characterize the liposomal particle size and zeta potential. DLS data were recorded using Laser Light Scattering Spectrometer (ALV/DLS/SLS-5022F, ALV/Laser Vertriebsgesellschaft m.b.H, German). Endocytosis inhibition experiments were performed using Flow Cytometry Analyzer (BD LSR Fortessa).

1.2 Synthesis

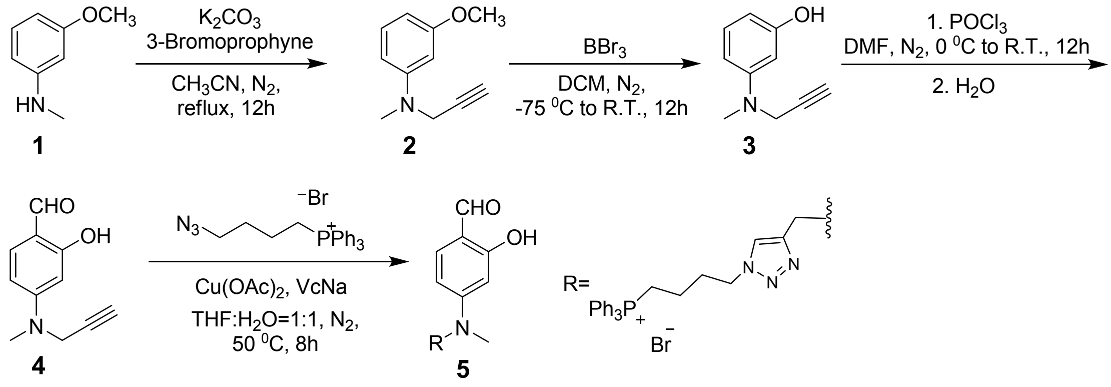

Compound 2 3-methoxy-N-methyl-N-(prop-2-ynyl)aniline

A reaction mixture of 3-methoxyaniline (2.0 g, 16.0 mmol), 3-bromoprophyne (1.9 g, 16.0 mmol) and K_2CO_3 (2.2 g, 16.0 mmol) in acetonitrile (50 mL) was refluxed under nitrogen for 12 h. After evaporation, the residue was extracted with CH_2Cl_2, washed and dried with anhydrous Na_2SO_4. Then the concentrated liquid was further purified by column chromatography to give yellow oil (2.1 g, 81%).

^{1}H NMR ($CDCl_3$, 400 M Hz): δ 2.17 (s, 1H), 2.94 (s, 3H), 2.78 (s, 3H), 4.01 (s, 2H), 6.40 (m, 2H), 6.46 (m, 1H), 7.17 (m, 1H);

^{13}C NMR ($CDCl_3$, 100 MHz) δ: 160.71, 150.47, 129.81, 107.23, 103.13, 100.94, 79.37, 72.15, 55.16, 42.47, 38.61.

Compound 3 3-(methyl(prop-2-ynyl)amino)phenol

Compound **2** (2.1 g, 12.0 mmol) was dissolved in 10 mL refreshed CH_2Cl_2, and boron tribromide (1.5 mL, 15.0 mmol) was added at -78 ℃. The mixture was warmed slowly to room temperature and stirred for 12 h. Cold methanol was added to quench extra boron tribromide. After evaporation and extraction, the residue was purified by column chromatography to give yellow oil (1.1 g, 56%).

^{1}H NMR ($CDCl_3$, 400 M Hz): δ 2.18 (s, 1H), 2.93 (s, 3H), 4.00 (s, 2H), 5.57 (s, 1H), 6.31 (m, 2H), 6.33 (d, 1H, J = 8.4 Hz), 7.11 (t, 1H, J = 16.2 Hz);

^{13}C NMR ($CDCl_3$, 100 MHz) δ: 160.94, 148.22, 130.27, 106.47, 103.29, 100.40, 80.01, 72.67, 53.39, 40.53.

Compound 4 2-hydroxy-4-(methyl(prop-2-ynyl)amino)benzaldehyde

$POCl_3$ (1 mL, 7.0 mM) was added slowly in anhydrous DMF (5 mL) in the ice-water bath and stirred for 30 min. Then compound **3** (1.1 g, 7.0 mmol) dissolved in DMF was added in drops. The mixture was slowly warmed to room temperature and stirred overnight. The reaction solution was poured into ice, stirred for a few minutes, and filtered to give light brown solid (830.0 mg, 63%).

^{1}H NMR ($CDCl_3$, 400 M Hz): δ 2.18 (s, 1H), 3.11 (s, 3H), 4.12 (d, 2H, J = 2.4 Hz), 6.23 (d, 1H, J = 2.4 Hz), 6.40 (dd, 1H, J_1 = 8.7 Hz, J_2 = 1.8 Hz), 7.36 (d, 1H, J = 9.0 Hz), 9.59 (s, 1H), 11.55 (s, 1H);

^{13}C NMR ($CDCl_3$, 100 MHz) δ: 192.94, 164.00, 155.13, 135.22, 112.64, 105.37, 98.76, 78.09, 77.39, 77.07, 76.75, 72.72, 41.74, 38.3

Compound 5

(4-(4-(((4-formyl-3-hydroxyphenyl)(methyl)amino)methyl)-1H-1,2,3-triazol-1-yl)butyl)triphenylphosphonium bromide

Compound 4 (178.0 mg, 1.1 mmol), (4-azidobutyl)triphenylphosphonium bromide(**PPh_3-N_3**, 570.0 mg, 1.3 mmol) and sodium ascorbate (837.7 mg, 4.2 mmol) were dissolved in THF and water (1:1). Then $Cu(OAc)_2{\cdot}H_2O$ (422.1 mg, 2.1 mmol) was added into the solution. The mixture was stirred at 80 °C for 8 h. The mixture was extracted with CH_2Cl_2, washed with water and dried with anhydrous Na_2SO_4. Then the concentrated liquid was further purified by column chromatography to give white solid (410.0 mg, 62%).

^1H NMR (CD_3OD, 400 M Hz):δ11.43 (1H,s), 9.46 (1H,s), 8.16(1H,s), 7.77(9H, m), 7.58(6H, m), 7.24(1H, s), 6.42(1H, dd, J1=9.2 Hz, J2=2.0 Hz), 6.12(1H, d, J=2.0 Hz), 4.27(2H, s), 3.86(2H, m), 3.08(2H, s), 2.30(2H, m), 2.23(2H, s), 1.56(2H, m);

^{13}C NMR ($CDCl_3$, 100 MHz):δ 192.32, 164.04, 155.43, 143.49, 135.10, 135.05, 135.02, 133.69, 133.59, 130.52, 130.40, 123.36, 118.49, 117.63, 112.13, 105.23, 97.79,, 48.72, 47.61, 38.75, 29.96,, 21.98, 21.47.

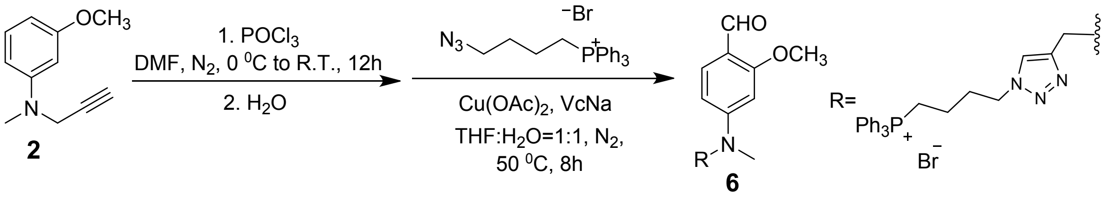

Compound 6

(4-(4-(((4-formyl-3-methoxyphenyl)(methyl)amino)methyl)-1H-1,2,3-triazol-1-yl)butyl)triphenylphosphonium bromide

$POCl_3$ (1 mL, 7.0 mM) was added slowly in anhydrous DMF (5 mL) in the ice-water bath and stirred for 30 min. Then compound **2** (1.1 g, 7.0 mmol) dissolved in DMF was added in drops. The mixture was slowly warmed to room temperature and stirred overnight. The reaction solution was poured into ice, stirred for a few minutes, and filtered to give olive solid. The olive product, (4-azidobutyl)triphenylphosphonium bromide (**PPh_3-N_3**, 570.0 mg, 1.3 mmol) and sodium ascorbate (837.7 mg, 4.2 mmol) were dissolved in THF and water (1:1). Then $Cu(OAc)_2{\cdot}H_2O$ (422.1 mg, 2.1 mmol) was added into the solution. The mixture was stirred at 80 ℃ for 8 h. The mixture was extracted with CH_2Cl_2, washed with water and dried with anhydrous Na_2SO_4. Then the concentrated liquid was further purified by column chromatography to give white solid (410.0 mg, 62%).

^1H NMR ($CDCl_3$, 400 MHz): δ10.14 (1H,s), 8.27 (1H,s), 7.67(9H, m), 7.63(7H, m), 6.39(1H, dd, J=9.2 Hz), 6.29(1H, s), 4.69(4H, m), 4.13(2H, m), 3.91(3H, s), 3.20(3H, s), 2.37(2H, m), 1.64(4H, m);

^{13}C NMR ($CDCl_3$, 100 MHz), δ (ppm): 187.50, 163.86, 155.14, 135.07, 135.04, 133.71, 133.61, 130.54, 130.41, 118.47, 117.62, 114.98, 104.94, 93.94, 55.54, 48.69, 47.95, 39.06, 30.02, 29.85, 22.07, 21.56, 19.18.

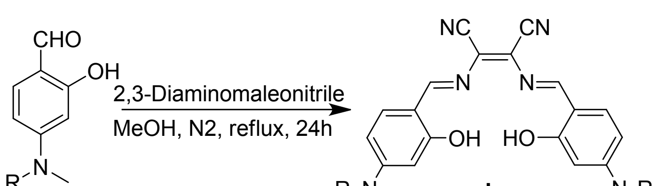

Compound L_1

A mixture of compound **6** (220.0 mg, 0.4 mmol) and diaminomaleonitrile (23.0 mg, 0.2 mmol) was dissolved in 10 mL MeOH and refluxed overnight in the presence of a drop of concentrated H_2SO_4. Solvent was removed by rotary evaporator, and the product **L_2** was recrystallized from DCM/ether as violet solid after filtration (100 mg, 39%).

^{1}H NMR (400 MHz, d^{6}DMSO): δ11.5(s, 2H), 8.03(s, 2H), 7.89-7.75(m, 30H), 7.59(m, 2H), 6.50(m, 1H), 6.42(m, 1H) 6.32(m, 1H) 6.17(m, 1H), 4.53(s, 4H), 4.05(s, 4H), 3.36(m, 4H), 3.10(s, 6H), 1.49(s, 4H), 1.14(m, 4H).

HR-ESI: $[M+H]^+$ calcd. for $[C_{70}H_{68}N_{12}O_2P_2]^{2+}$, 585.25406; found, 585.25262.

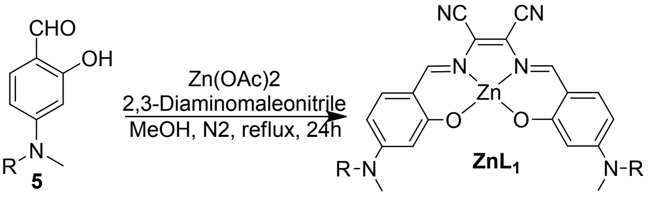

Compound ZnL_1

ZnL_1 was prepared according to the procedure described in the literature.[1]

A mixture of compound **5** (220.0 mg, 0.4 mmol) and diaminomaleonitrile (23.0 mg, 0.2 mmol), and $Zn(OAc)_2 \cdot 2H_2O$ (47.3 mg, 0.2 mmol) was dissolved in 50 mL MeOH and refluxed overnight. Most of the solvent was removed by rotary evaporator, and the product **ZnL_1** was obtained as dark violet solid after filtration (180.0 mg, 71%).

^{1}H NMR (d^{6}DMSO, 400 M Hz): δ 3.20 (s, 3H), 3.37 (s, 12H), 3.54 (m, 4H), 3.71 (m, 2H), 3.88 (m, 2H), 4.77 (s, 2H), 5.60 (d, 1H, J = 9.2 Hz), 6.55 (d, 1H, J = 8.8 Hz), 7.44 (d, 1H, J = 8.8 Hz), 8.15 (s, 1H), 8.56 (s, 1H), 9.56 (s, 1H);

HR-ESI: $[M+H]^+$ calcd. for $[C_{70}H_{66}N_{12}O_2P_2Zn]^{2+}$, 616.21222; found, 616.20937.

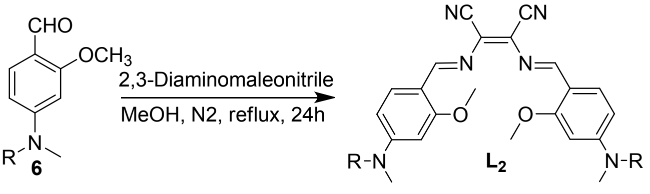

Compound L_2

A mixture of compound **6** (50 mg, 0.088 mmol) and diaminomaleonitrile (5.0 mg, 0.2 mmol) was dissolved in 10 mL MeOH and refluxed for 8 h. Solvent was removed by rotary evaporator, and the product **L_2** was recrystallized from methanol and ether as dark red solid after filtration (29 mg, 51%).

^{1}H NMR (d^{6}DMSO, 400 M Hz): δ 8.90(s, 2H), 7.90(s, 2H), 7.82-7.50(m, 30H), 7.48(s, 2H), 6.32(s, 2H), 6.22(s, 2H), 4.67(s, 4H), 4.46(s, 4H), 3.87(s, 6H), 3.36(m, 4H), 3.17(s, 6H), 2.16(s, 4H), 1.58(s, 4H);

HR-ESI: $[M+H]^+$ calcd. for $[C_{72}H_{72}N_{12}O_2P_2]^{2+}$, 599.26767; found, 599.26827.

1.3 Quantum yield determination

Quantum yields of one photon emission of $\mathbf{L_1}$, $\mathbf{H_2L_1}$, $\mathbf{L_2}$ were measured with Rhodamine B as reference (Φ=0.65).[2] The one photon fluorescence measurements were performed in 1cm quartz cells with 1 μM compound in DMSO on a fluorescence lifetime and steady state spectrophotometer (Edinburgh Instrument FLS920) equipped 450 W Xenon light, slits 2.5 × 2.5. The values of fluorescence quantum yield, Φ (sample), were calculated according to equation as following[3]:

$$\frac{\Phi_{sample}}{\Phi_{ref}} = \frac{OD_{ref} I_{sample} d^2_{sample}}{OD_{sample} I_{ref} d^2_{ref}} \quad (Equation.\ \mathrm{S1})$$

I: integrated emission intensity.

OD: optical density at the excitation wavelength.

d: the refractive index of solvents. d_{DMSO}=1.478; $d_{Ethanol}$=1.361

1.4 Two photon absorption

The two photon absorption spectra of ZnL_1 and L_1 were determined over a broad spectral region by the typical two photon induced fluorescence method relative to Rhodamine B as standard. The two photon fluorescence data were acquired using a Tsunami femtosecond Ti: Sapphire laser (pulse width ≤100fs, 80 MHz repetition rate, tuning range 720–870 nm Spectra Physics Inc., USA). The two photon fluorescence measurements were performed in a 1cm quartz cell with 40 μM Salen in DMSO and the excitation power density is set to be 100 mW. The quadratic dependence of two photon induced fluorescence intensity on the excitation power was verified (from 34 mW to 127 mW) for excitation wavelength at 840 nm. The two photon absorption cross section of the Salens (δ_{sample}) was calculated at each wavelength according to equation as following[4] :

$$\frac{\delta_{sample}}{\delta_{ref}} = \frac{\phi_{ref} C_{ref} I_{sample} d_{sample}}{\phi_{sample} C_{sample} I_{ref} d_{ref}} \quad (Equation.\ \mathrm{S2})$$

Φ: quantum yield of sample and reference.

I: integrated emission intensity.

C: concentration of each sample.

d: the refractive index of solvents. d_{DMSO}=1.478; $d_{Ethanol}$=1.361

1.5 Lop P determination

Equal amounts of n-octanol and water were thoroughly mixed by an oscillator for 24 h. The mixture was then left to separate for another 24 h to finally yield water and octanol phase, each saturated with the other. Each complex was dissolved in water (C_o) and water saturated with octanol to form a 20 μM solution. Then the latter was mixed with equal amounts of octanol (saturated with water) and shaken again as described above. After separation, the final concentrations of compounds in water corresponded to C_w. The final concentration in 1-octanol corresponded to $C_{oil.}$ [5]

$$P = \frac{C_{oil}}{C_{water}} \quad (Equation.\ \mathrm{S3})$$

1.6 The Mitochondria Isolation

Mitochondrial permeation capabilities more exact using freshly isolated mitochondria. To prepare mitochondrial fractions, HeLa cells were harvested and the pellet processed using a Mitochondrial Isolation Kit for Cultured Cells (BiYunTian company). All isolation operations were completed under sterile conditions at 4 °C.

1.7 GUV preparation

GUVs were prepared by gentle hydration method. [6] Lipids dissolved in chloroform were mixed at designated ratios in a glass flask with a final mass of 1 mg. The chloroform was evaporated under reduced pressure distillation followed by storage overnight. The dried lipids film was stored at -20 °C prior to use. To hydrate the lipid, 1 mL buffer PBS 8.0 buffer was added to the film for about an hour after prehydration. The liposome size and zeta-potential were measured using Zeta Plus. Measurements were performed by dispersing liposomes at a concentration of 1 mg mL^{-1} in buffer pH 8.0. The temperature was set at 25 °C for all measurements. ZnL_1 and L_1 solution were added into the well prepared GUVs solution, and the mixture was stirred softly for half an hour. To get confocal images, the vesicles were filled with sucrose and were in a glucose environment at osmolarity 200 mOsm g^{-1}. The effect of gravity deforming the vesicle is visible from the snapshot.

1.8 Cell culture

All HeLa cells were incubated in complete medium (Dulbecco's modified Eagle's Medium, supplemented with 10% fetal bovine serum (FBS) and 1% penicillin-streptomycin) at 37 °C in atmosphere containing 5% CO_2.

1.9 Colocalization assay

HeLa cells were placed onto 0.1 mM poly-D-lysine coated glasses in complete media and the cells were incubated for 24 h. A stock solution of Salens in chromatographic grade, anhydrous DMSO was prepared as 2 mM. The solution was diluted to a final concentration of 2 μM by complete growth medium. Stock solutions of Lyso Tracker Green DND-26, MitoTracker Green FM were prepared as 1 mM, and the stock solution was diluted to the working concentrations in complete medium (Lyso Tracker: 75 nM, Mito Tracker: 100 nM).

Transfection with EHD1-EGFP, and FYVE-EGFP plasmids: Hela cells were grown to about 80% confluency and then reseeded in 24-well plates; cells were transfected with 0.8 μg plasmids, using LipfectamineTM 2000 according to manufacturer's instruction. After incubation of 2 μM Salens for different period of time, cells were washed with PBS buffer twice before confocal experiments. Images were taken under conditions as follows: 60× immersion lens with a resolution of 1024×1024 and a speed of 0.5 frame per second, 543 nm excitation wavelength and 552 to 617 nm detector slit, 100% laser power for dye, and 80% laser power for LysoTracker (ex: 488 nm, em: 505-560 nm), MitoTracker (ex: 488 nm, em: 505-560 nm), FYVE-EGFP (ex: 488 nm, em: 505-560 nm), EHD1-EGFP (ex: 488 nm, em: 505-560 nm). Differential interference contrast (DIC) and fluorescent images were processed and analyzed using ImageJ. The Pearson's Coefficient was calculated by ImageJ.

1.10 Cell uptake mechanism assay

The cellular uptake of luminescent metal complexes is primarily examined using two complementary methods, flow cytometry and confocal microscopy. In the temperature effect assay, cells were placed at 4 ^{o}C for 15 minutes, and then incubated with **ZnL_1** in absence or presence of pyridine for another hour at 4 ^{o}C. For endocytosis mechanism investigation, various endocytosis inhibitors including chlorpromazine (inhibitor of clathrin-mediated endocytosis), genistein (inhibitor of caveolae-mediated endocytosis), cytochalasin D (inhibitor of macropinocytosis) were applied to cells for 30 minutes. Then medium containing both inhibitors and complex was used for incubation for another hour. For flow cytometry, cells are detached from culture either before or after incubation with the metal complex to produce a cell suspension. Untreated cells are used for as a control for autofluorescence. The cells are inspected individually as they pass single file through the laser beam of 561 nm and the instrument records their light scatter and luminescence from 570 to 610 nm. The distribution of luminescence for the cell population is depicted as a histogram of the number of cells versus luminescence intensity.

In the membrane potential effect assay, HeLa cells were detached from culture and washed three times with either HBSS (containing 5.8 mM K^+) or high K^+-HBSS (containing 170 mM K^+). Some of the cells in HBSS were pretreated with 10 μM nigericin for 30 min at 37°C. The cells were incubated with 2μM **ZnL_1** for 1h at 37 °C in one of the following solutions: HBSS, HBSS with nigericin (to hyperpolarize the cells), or high K^+-HBSS (to depolarize the cells). After incubation, the cells were rinsed, and the extent of uptake was analyzed by confocal imaging and dealt with ImageJ. Images were taken under conditions as follows: 60× immersion lens with a resolution of 1024×1024 and a speed of 0.5 frame per second, 543 nm excitation wavelength and 552 to 617 nm detector slit, 100% laser power for dye. Differential interference contrast (DIC) and fluorescent images were processed and analyzed using ImageJ.

In the Organic Cation Transporter effect assay, HeLa cells were detached from culture and washed three times with HBSS (containing 5.8 mM K^+), and then pretreated for 20 min with either 1 mM cation transport inhibitor (Tetrabutylammonium bromide). The cells were then incubated with 2μM **ZnL_1** for 1h at 37 °C in one of the following solutions: HBSS, HBSS with 1 mM cation transport inhibitor (Tetrabutylammonium bromide). After incubation, the cells were rinsed, and the extent of uptake was analyzed by confocal imaging and dealt with ImageJ. Images were taken under conditions as follows: 60× immersion lens with a resolution of 1024×1024 and a speed of 0.5 frame per second, 543 nm excitation wavelength and 552 to 617 nm detector slit, 100% laser power for dye. Differential interference contrast (DIC) and fluorescent images were processed and analyzed using ImageJ.

1.11 Estimation of aggregation constants of ZnL_1

The aggregation constant for $\mathbf{ZnL_1}$ was calculated by pyridine titration at 298K. The basic method were according to Kleij`s work[7]. 0-350 000 equiv. pyridine were added to a water solution of 20 μM $\mathbf{ZnL_1}$. The reaction and correlative equilibrium constants are shown as equation (1):

$$\mathbf{(ZnSalen)_n + nPy \rightleftharpoons n\ ZnSalen(Py)} \quad K_1 = K^n_{py}/K_{agg} \quad K_{agg} = K^{n-1}_{n/n+1} \qquad (1)$$

K_{agg} and $K_{n/n+1}$ represent the overall aggregation constant and stepwise aggregation constant, as shown in equation (2) and (3):

$$\mathbf{n\ ZnSalen \rightleftharpoons (ZnSalen)_n} \qquad K_2 = K_{agg} \qquad (2)$$

$$\mathbf{ZnSalen + (ZnSalen)_n \rightleftharpoons (ZnSalen)_{n+1}} \qquad K_3 = K_{n/n+1} \qquad (3)$$

The equilibrium constant K_1 was determined by fitting for $A_{590\ nm}$ and equiv. of pyridine.

For each $A_{590\ nm}$ obtained from the titration, there was relationship as

$$A = \varepsilon_1 c_1 + \varepsilon_2 c_2 \qquad (4)$$

$$nc_1 + c_2 = c_{total} \qquad (5)$$

A is the absorbance at 590 nm; n is the aggregation number of $\mathbf{ZnL_1}$ aggregates; ε_1 and ε_2 are the molar extinction coefficient of $\mathbf{ZnL_1}$ aggregates and its pyridine complex in H_2O, which are obtained from the limit value of fitting curve; c_1, and c_2 represent the concentration of $\mathbf{ZnL_1}$ aggregates, $\mathbf{ZnL_1}$-pyridine complex, respectively; and c_{total} is the total concentration of ZnSalen species, which is 20 μM in this case. Combining equation (4) and (5), c_1 and c_2 could be represented as

$$c_1 = \frac{c_{total}}{n} \bullet \frac{A-A_2}{A_1-A_2} \qquad (6)$$

$$c_2 = c_{total} \bullet \frac{A_1-A}{A_1-A_2} \qquad (7)$$

And the equilibrium constant of equation (1) could be written as

$$K_1 = (c_2)^n/(c_1 c_{py}) = \frac{n\, c_{total}^{n-2} (A_1-A)^n}{q(A-A_2)\,(A_1-A_2)^{n-1}} \qquad (8)$$

c_{py} is the concentration of pyridine, which depends on the equiv. of pyridine q; A_1 and A_2 are the limit absorbance of fitting curve, representing $A_{590\ nm}$ of totally aggregated and dissociated states, respectively.

To estimate K_{agg} and $K_{n/n+1}$, the coordination constant of pyridine was measured first. 0-40 equiv. pyridine were added to the 20 μM **ZnL₁** dichloromethane solution. As shown in equation (7), K_{py} was calculated by the curves of $A_{587\ nm}$ and equiv. of pyridine.

$$\textbf{ZnSalen} + \textbf{Py} \rightleftharpoons \textbf{ZnSalen(Py)} \qquad K_4 = K_{py} = \frac{c_4}{c_3\ c_{py}} \qquad \textbf{(9)}$$

c_3 and c_4 are the concentration of **ZnL₁** and its pyridine complex in dichloromethane solution, which can also be represented as

$$c_3 = c_{total} \cdot \frac{A-A_4}{A_3-A_4} \qquad \textbf{(10)}$$

$$c_3 = c_{total} \cdot \frac{A_3-A}{A_3-A_4} \qquad \textbf{(11)}$$

A is the absorbance at 587 nm for each titration; A_3 and A_4 are the absorbance of **ZnL₁** without pyridine coordination and totally corrdination, obtained from inital spectra and the limit value from data fitting, respectively. Thus, K_{agg} and $K_{n/n+1}$ could be determined by K_1, K_{py}, and n, according to equation (1).

1.12 DOSY experiments and estimation of molecular mass of ZnL₁

The DOSY spectroscopic were recorded using a Bruker-500 NMR. The calculation of molecular mass was according to Bella's report[8]. The molecular mass of two species is followed by

$$m_B = m_A\,(D_A/D_B)^2 \qquad \textbf{(12)}$$

Different amount of D_2O were added to 2 mM **ZnL₁** dissolved in DMSO-*d*^{6} solution. The ^{1}H signal of HDO was used as the reference, with molecular mass of 19 Da. Diffusion coefficient of **ZnL₁** was averaged by diffusion coefficients of all peaks on salen. Thus, the molecular mass of **ZnL₁** species in these condition could be estimated using equation 12.

2. Supplementary Tables

Table S1. Photophysical properties of **ZnL_1**, **L_1** and **L_2**.

Compound[a]	λ_{max} (nm, ε*10^4) [b]	λ_{em} (nm, λ_{ex}=380 nm)	Φ_F [c]
**ZnL_1**	384 (3.83), 434 (2.32), 589 (4.68)	629	0.26
**L_1**	437 (2.64), 567 (3.13)	631	0.29
**L_2**	434(0.98), 567(3.84)	624	0.21

[a] Compounds were dissolved in DMSO (20 μM). [b] ε: extinction coefficients, 10^4 $M^{-1}cm^{-1}$. [c] Quantum yields were measured using Rhodamine B in ethanol as reference.

Table S2. Lipophilicity (log $P_{o/w}$) of **ZnL_1**, **L_1** and **L_2**.

Compound	**ZnL_1**	**L_1**	**L_2**
Log$P_{o/w}$	0.12	-0.77	0.09

Table S3. Overall aggregation constant K_{agg}, stepwise aggregation constant $K_{n/n+1}$ of **ZnL_1**, at 298 K for an given aggregation number n.

n	5	6	7	8	9
K_{agg} (M^{1-n})	5.7×10^{42}	9.1×10^{52}	1.5×10^{63}	2.6×10^{73}	4.6×10^{83}
$K_{n/n+1}$(M^{-1})	4.9×10^{10}	3.9×10^{10}	3.4×10^{10}	3.1×10^{10}	2.9×10^{10}

Table S4. Diffusion coefficients (D) and corresponding estimated molecular mass (m) of **ZnL_1** at 30 °C in DMSO-*d^6* containing different amounts of D_2O.

contents of D_2O	D (**ZnL_1**) ($\times10^{-11}$ m^2s^{-1})	D (HDO) ($\times10^{-11}$ m^2s^{-1})	m (Da)
10%	9.22	75.1	1261
30%	6.61	60.7	1602

3. Supplementary Figures

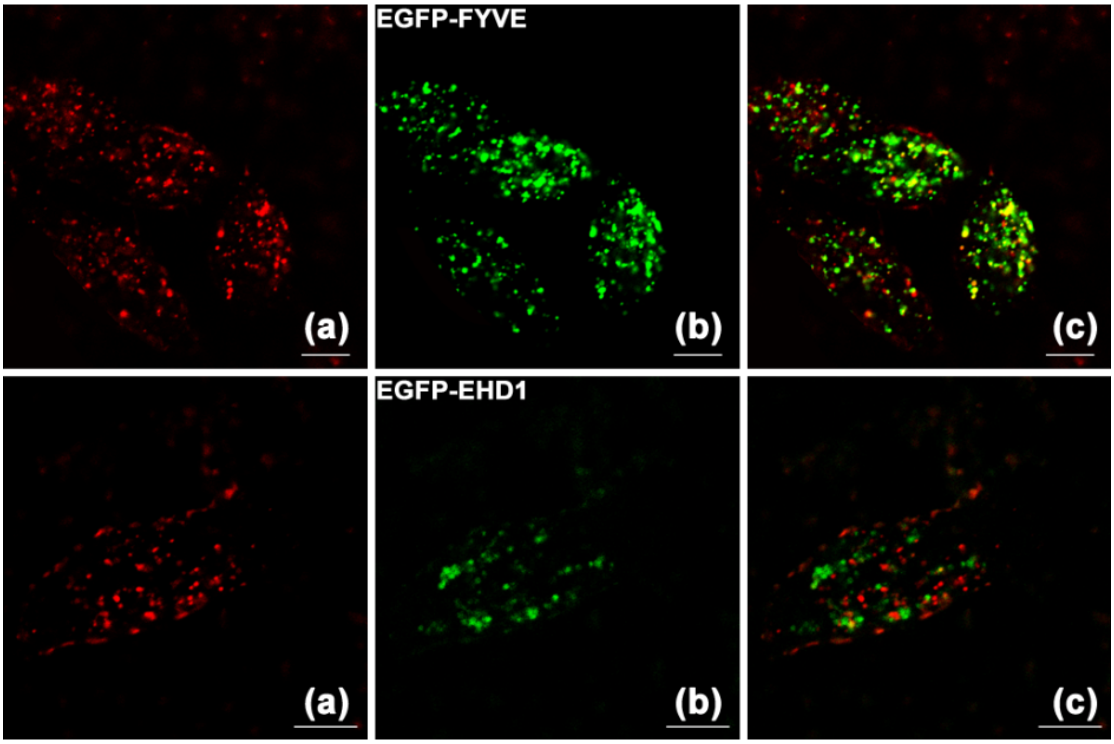

Figure S1. Fluorescence colocalization images of HeLa cells incubated with $\mathbf{ZnL_1}$ and endosome trackers: EGFP-FYVE (early endosome tracker, Pearson's coefficient: 0.53±0.06) or EGFP-EHD1 (late endosome tracker, Pearson's coefficient: 0.45±0.04). (a) Confocal fluorescence image of salens. (b) Confocal fluorescence image of trackers. (c) Merged image of (a) and (b).

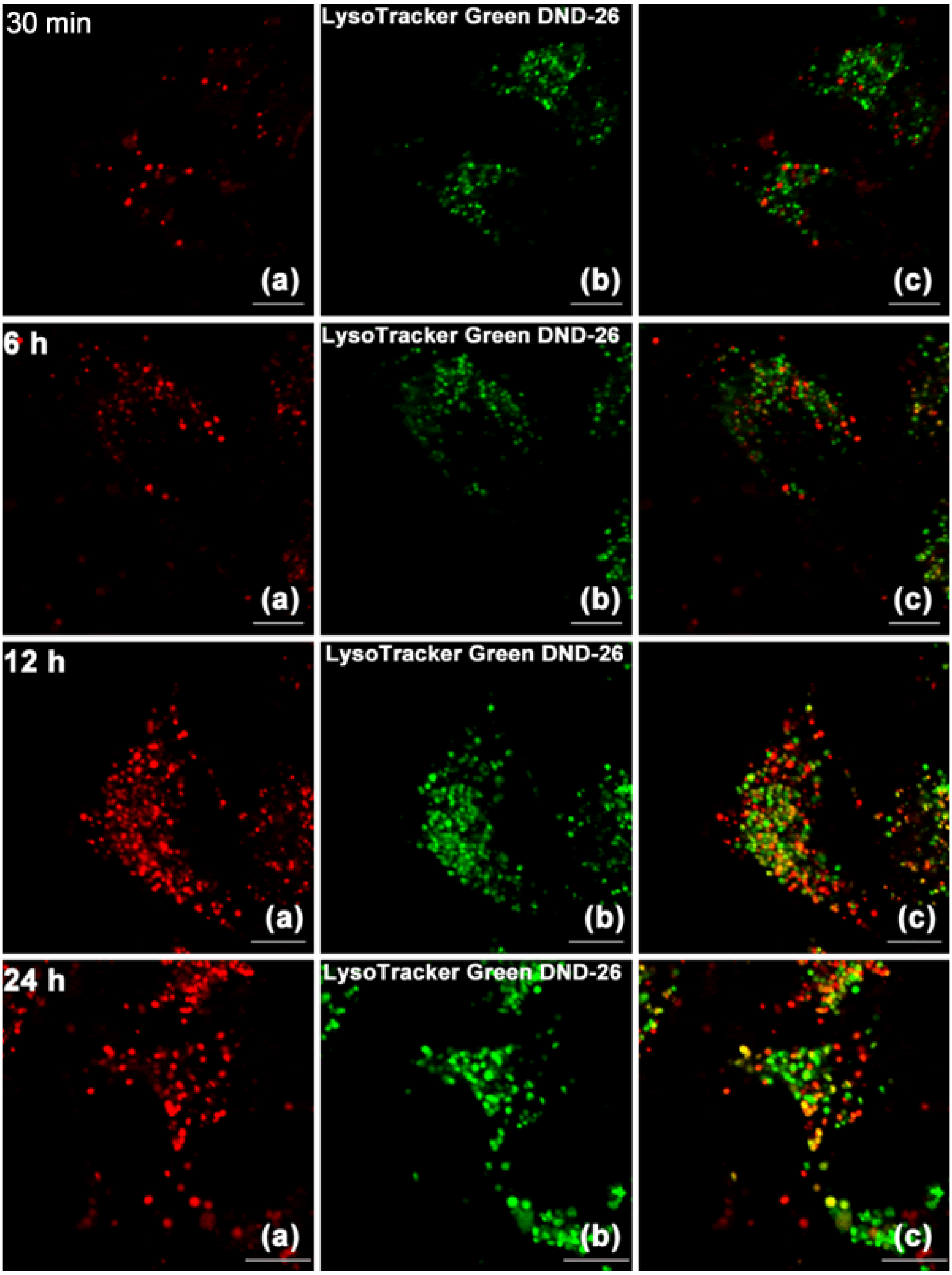

Figure S2. Time Tracing of cellular behaviors of $\mathbf{ZnL_1}$. (a) Confocal fluorescence image of Znsalen-di; (b) Confocal fluorescence image of LysoTracker Green DND-26; (c) Merged image of (a) and (b).

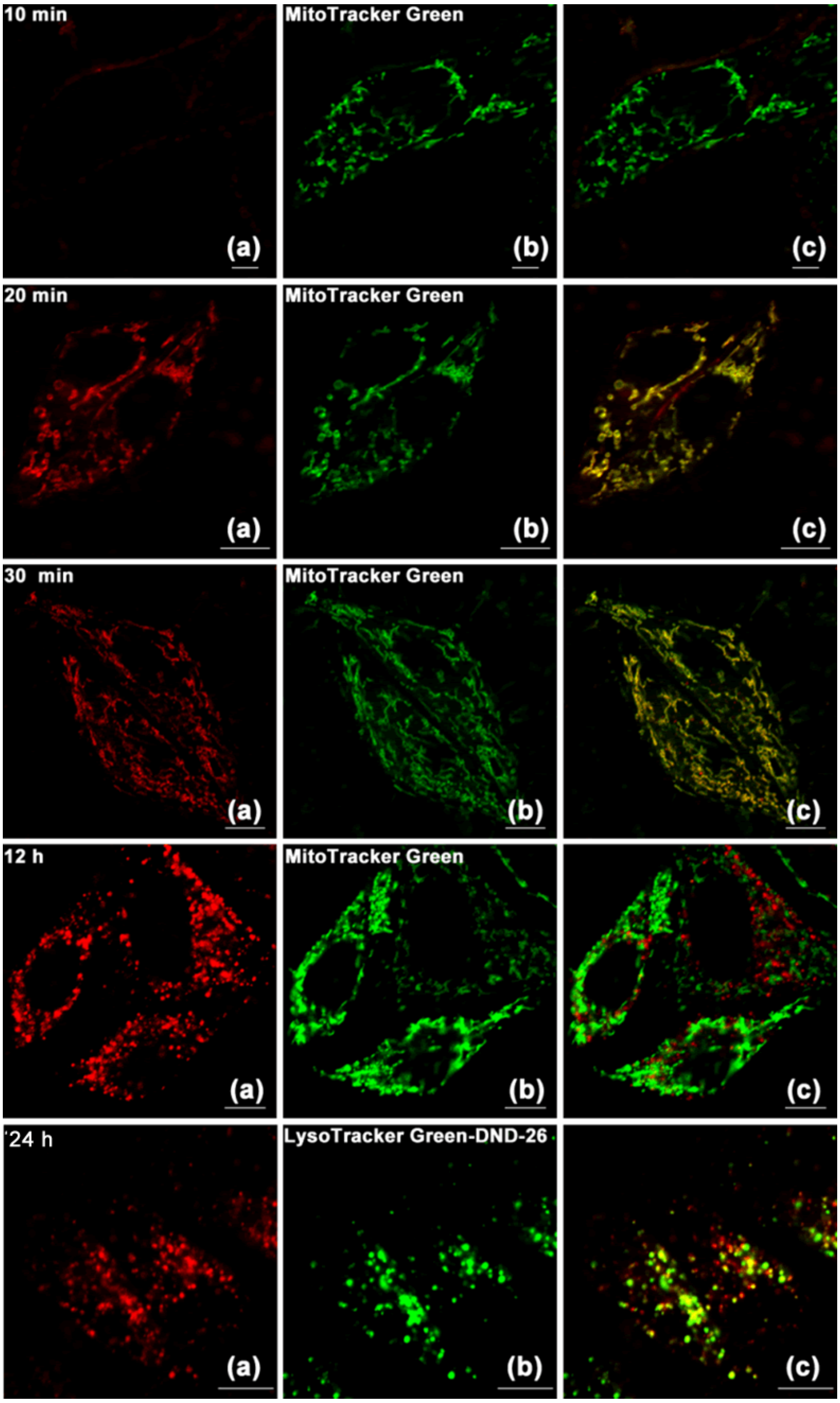

Figure S3. Time tracing of cellular behaviors of $\mathbf{L_1}$. (a) Confocal fluorescence image of $\mathbf{L_1}$; (b) Confocal fluorescence image of MitoTracker Green in the first four rows or LysoTracker Green DND-26 in the last row; (c) Merged image of (a) and (b).

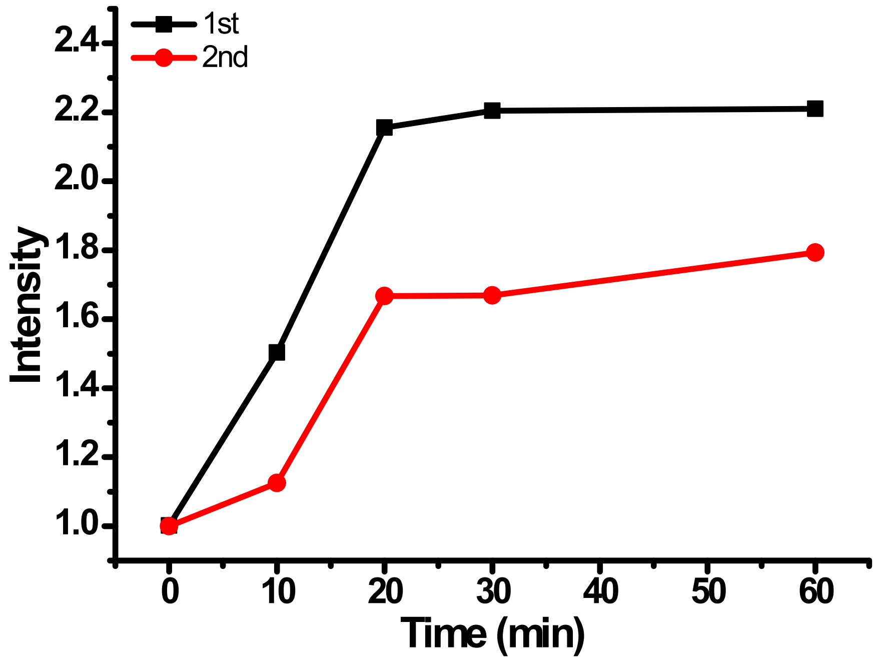

Figure S4. Mitochondrial potential confirmation using indicator JC-1. The aggregates were excited at 560 nm, and emission was collected at 590 nm.

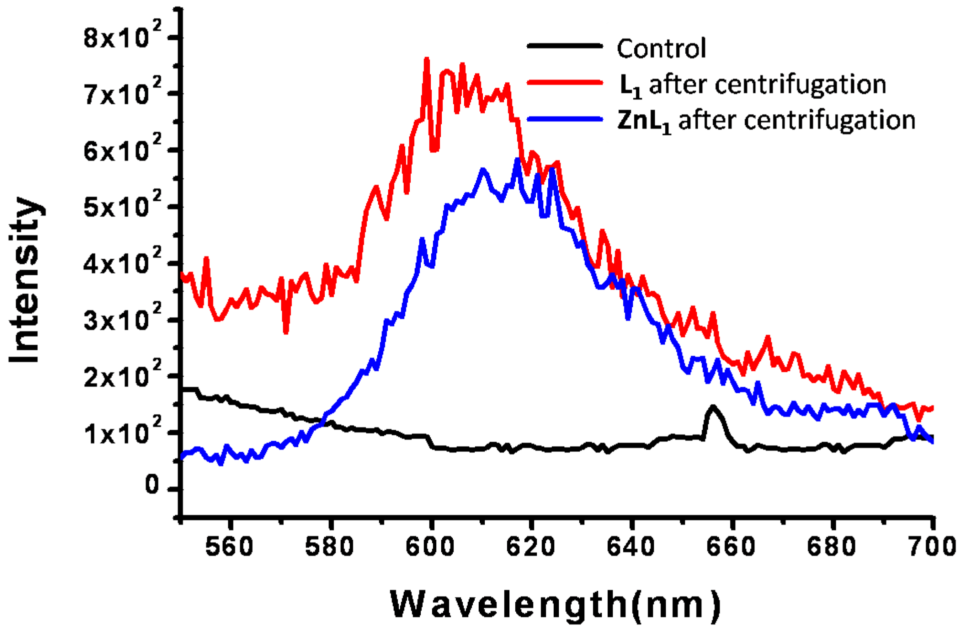

Figure S5. Mitochondrial permeability investigation of $\mathbf{ZnL_1}$ and $\mathbf{L_1}$. 2 μM of $\mathbf{ZnL_1}$ and $\mathbf{L_1}$ were incubated in mitochondria stock solution for 30 min. The mixture was resuspended for fluorescence detection after centrifugation.

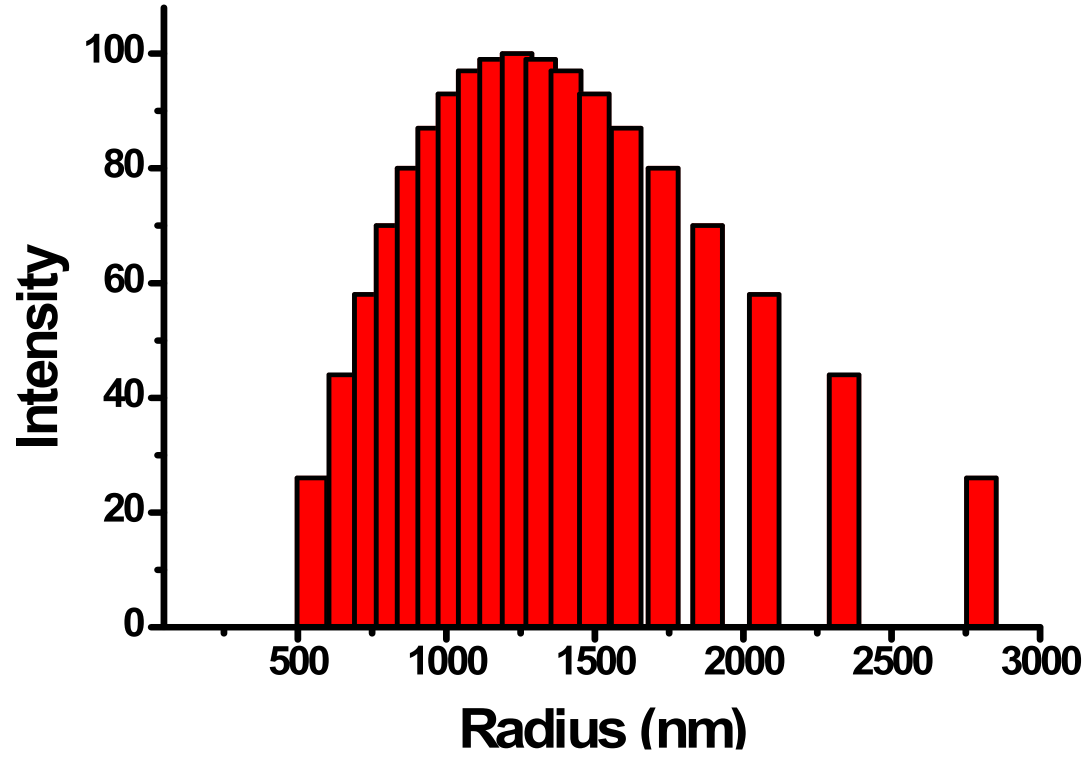

(a)

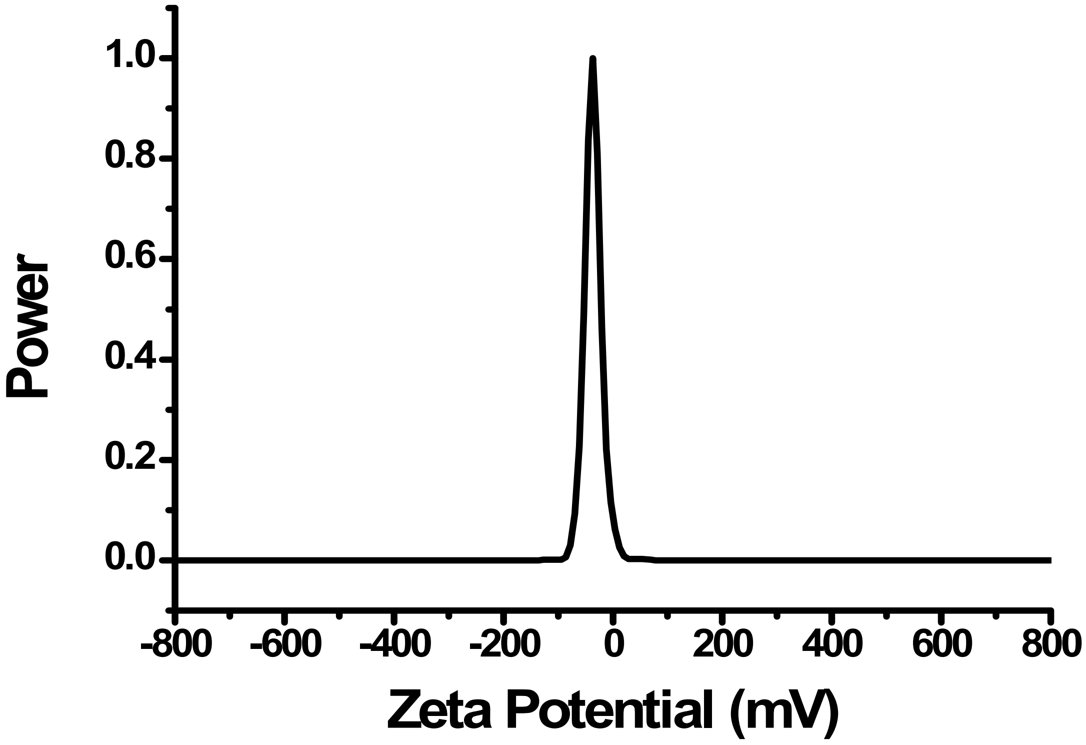

(b)

Figure S6. GUV characterization: (a) vesicle size characterization through particle analyzer: 2510±359 nm. (b) zeta potential examine of vesicles: -31.33±2.08 mV .

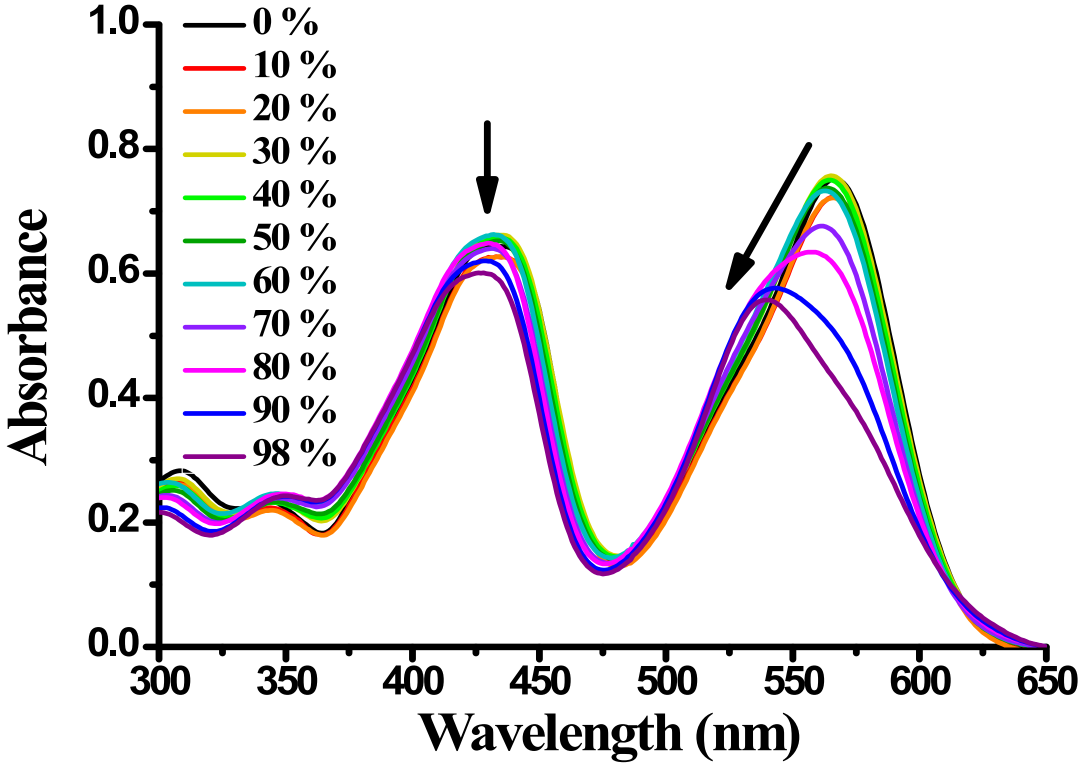

Figure S7. UV-*vis* spectra $\mathbf{L_1}$ (20 μM) in the mixed solution of H_2O/DMSO. The contents of H_2O was from 0% to 98%.

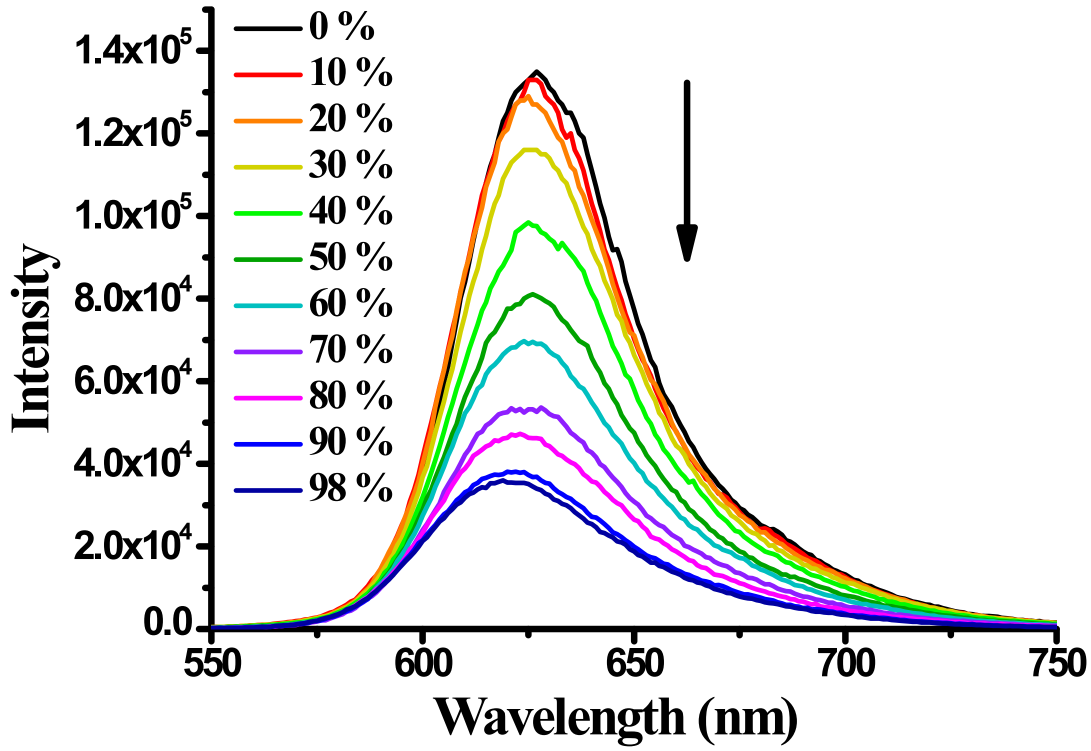

Figure S8. Emission spectra of $\mathbf{L_1}$ (40 μM) in mixed solution of H_2O/DMSO (v/v) (excitation wavelength: 380 nm). The contents of H_2O was from 0% to 98%.

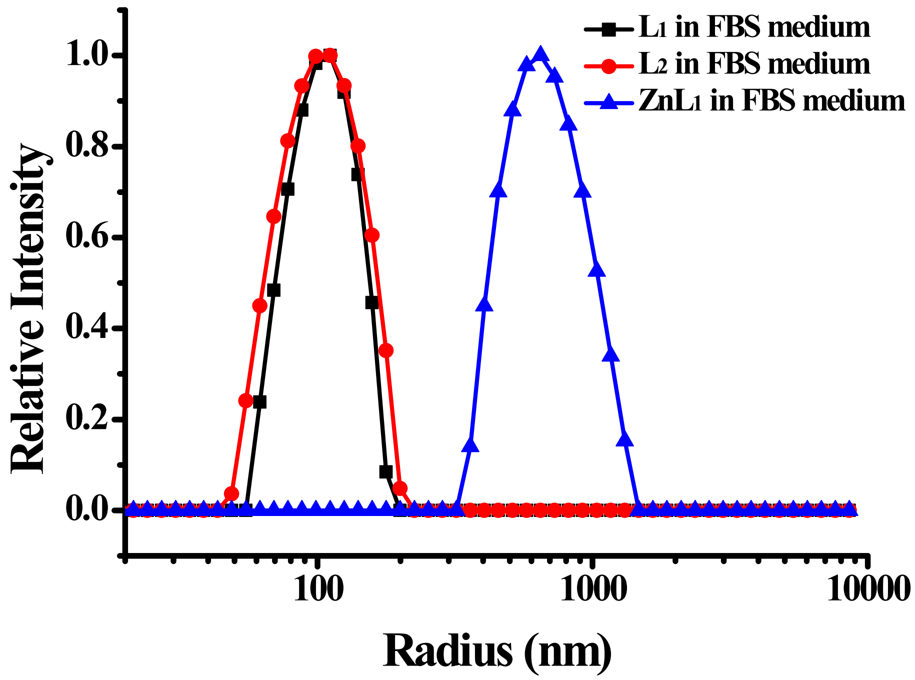

Figure S9. DLS analysis of **ZnL_1**, **L_1** and **L_2** in FBS medium. All complexes were dissolved into water with the assistance of 0.1% DMSO to a final concentration of $1*10^{-6}$ M.

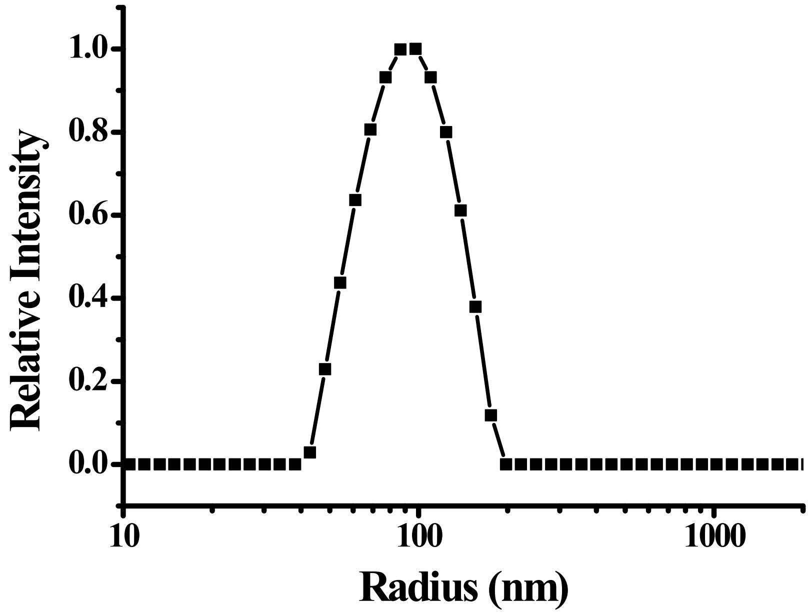

Figure S10. DLS analysis of $\mathbf{L_2}$ in water containing 1 % DMSO.

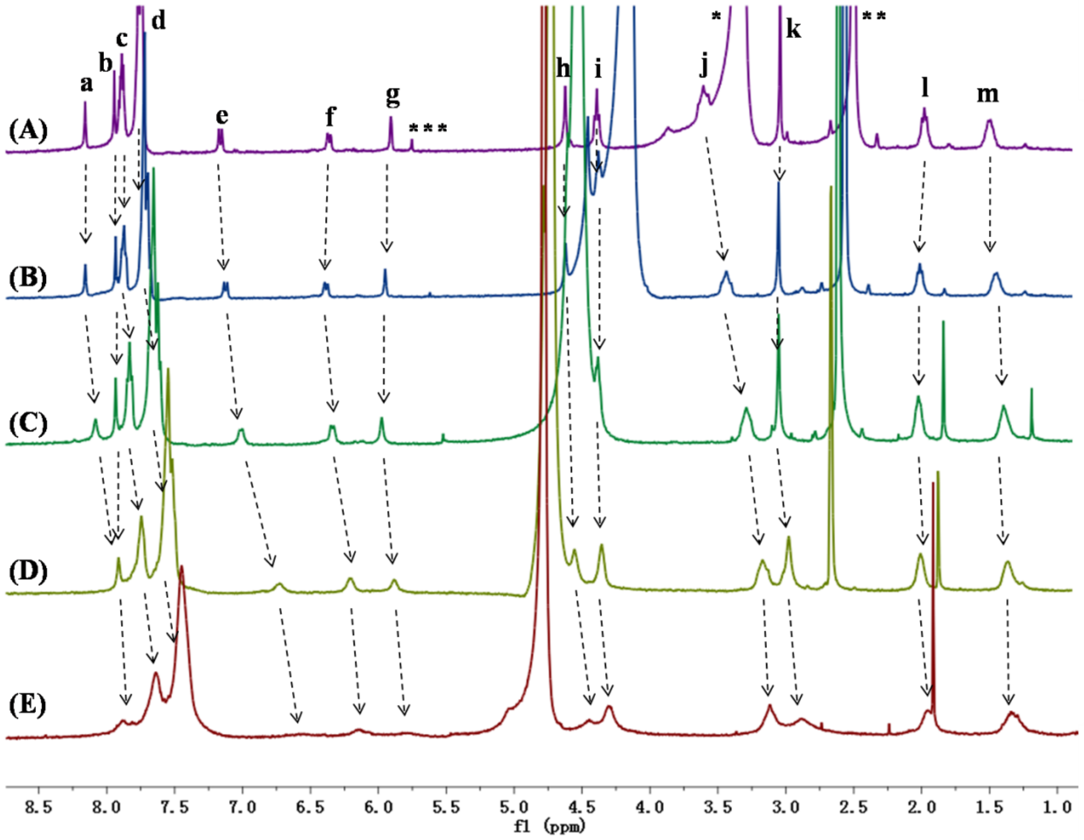

Figure S11 ^{1}H NMR of **ZnL$_1$** in DMSO-d^6/D_2O mixed solvents. The content of DMSO-d^6 is (A) 100%; (B) 70%; (C) 50%; (D) 20%; and (E) 0%. The Peaks labeled by *, **, and *** was H_2O, DMSO-d^5, and remained dichloromethane.

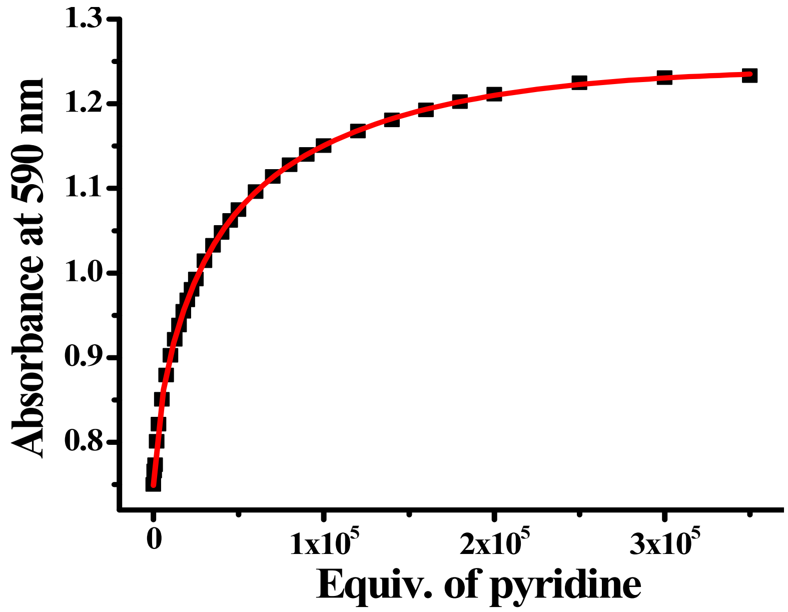

(a)

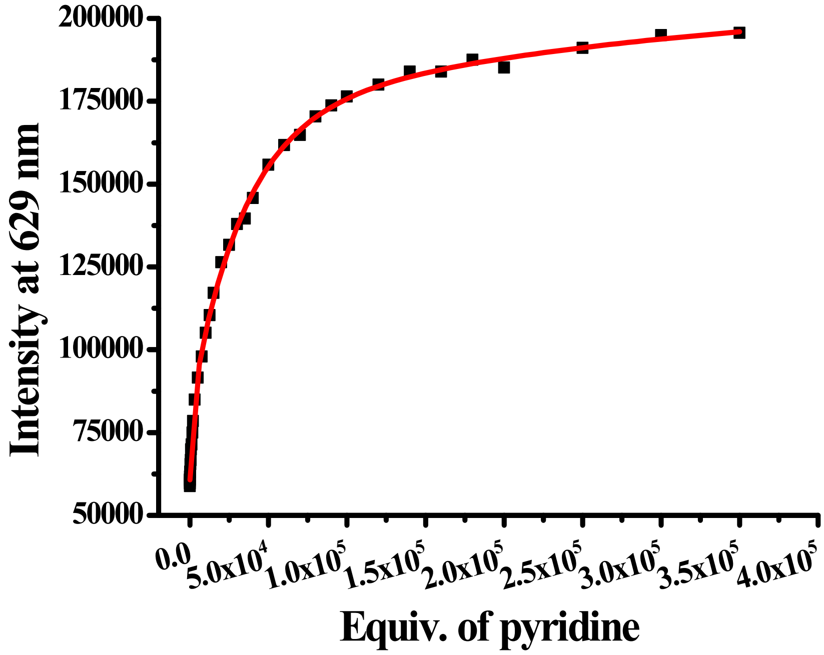

(b)

Figure S12. The plots of (a) absorption and (b) emission at 629 nm by titrating different amounts of pyridine into 20 μM **ZnL$_1$** water solution.

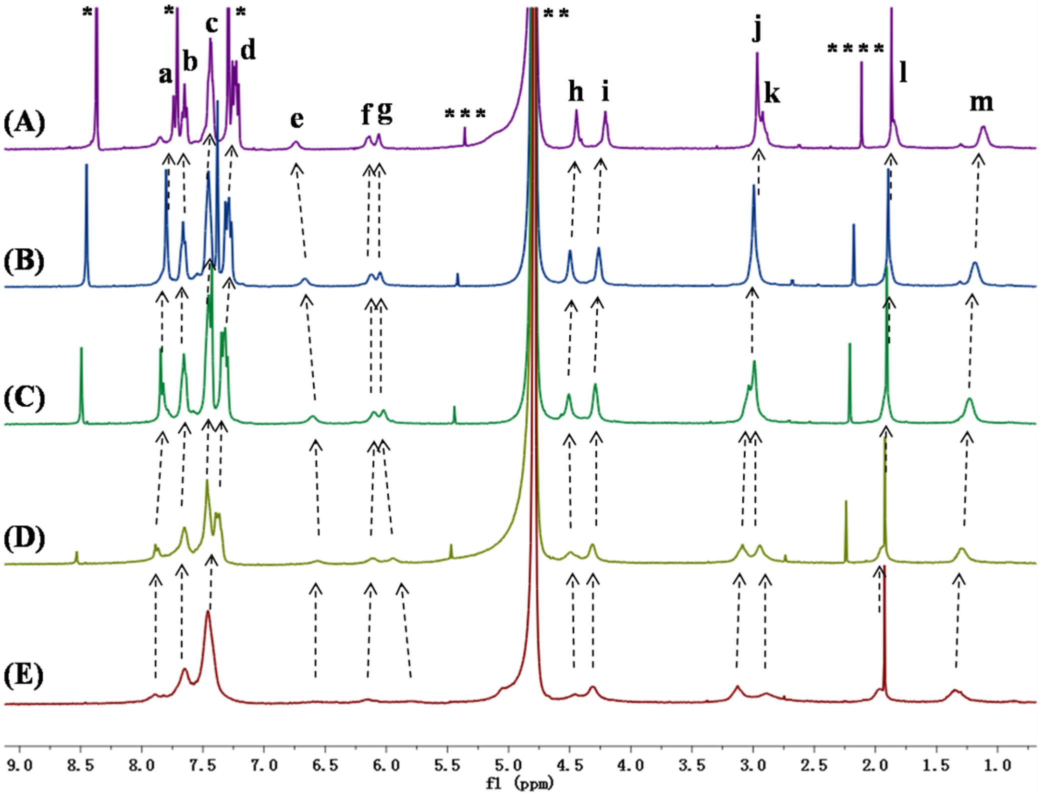

Figure S13 ^{1}H NMR of **ZnL$_1$** in D_2O and adding different equiv. of pyridine-*d*^{5}. The peaks labeled with stars are the peaks of pyridine-*d*^{5} in D_2O. (A) 928 equiv., (B) 464 equiv., (C) 232 equiv., (D) 58 equiv, (E) 0 equiv. Peaks labeled with *, **, ***, and **** were the remained peaks of pyridine-*d*^{5}, D_2O, dichloromethane, and acetone.

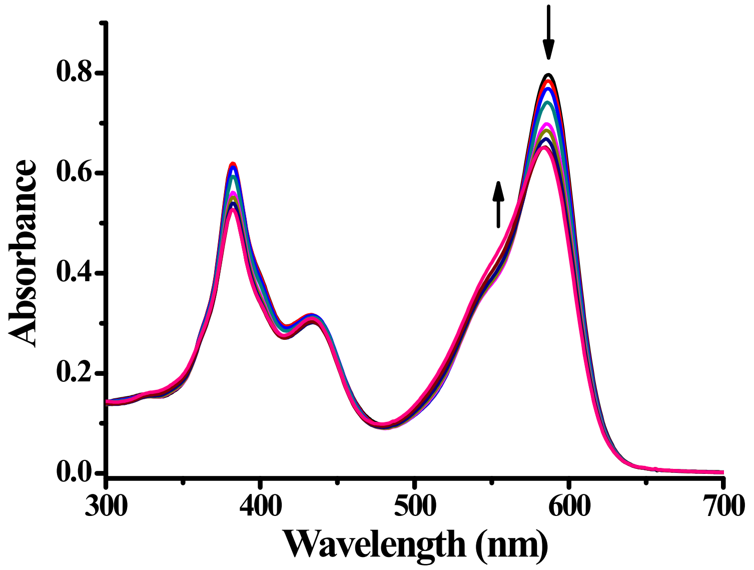

(a)

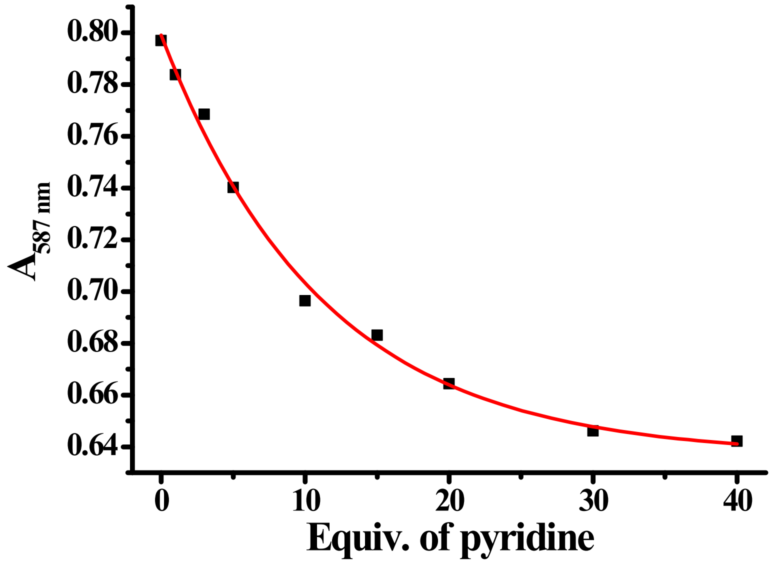

(b)

Figure S14 (a) Spectral changes of 20 μM **ZnL₁** by adding pyridine in dichloromethane, and (b) the trace of absorbance at 587 nm.

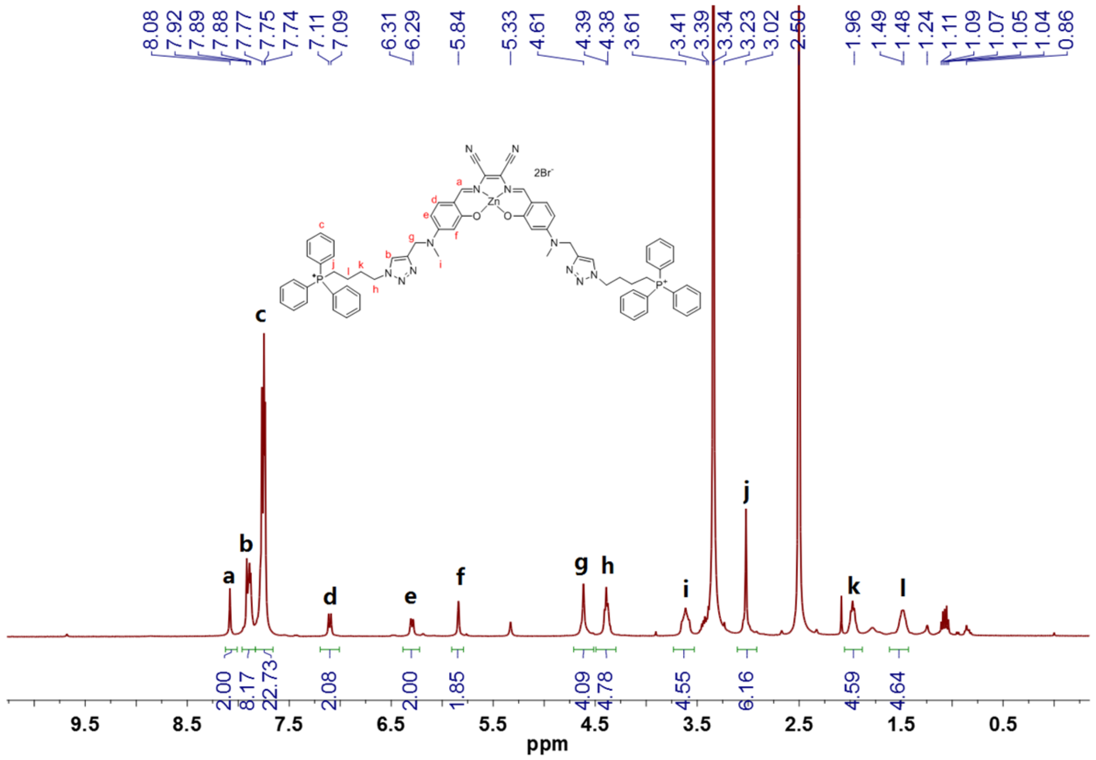

Figure S15. ^{1}H NMR spectrum of **ZnL_1** in DMSO-d^6.

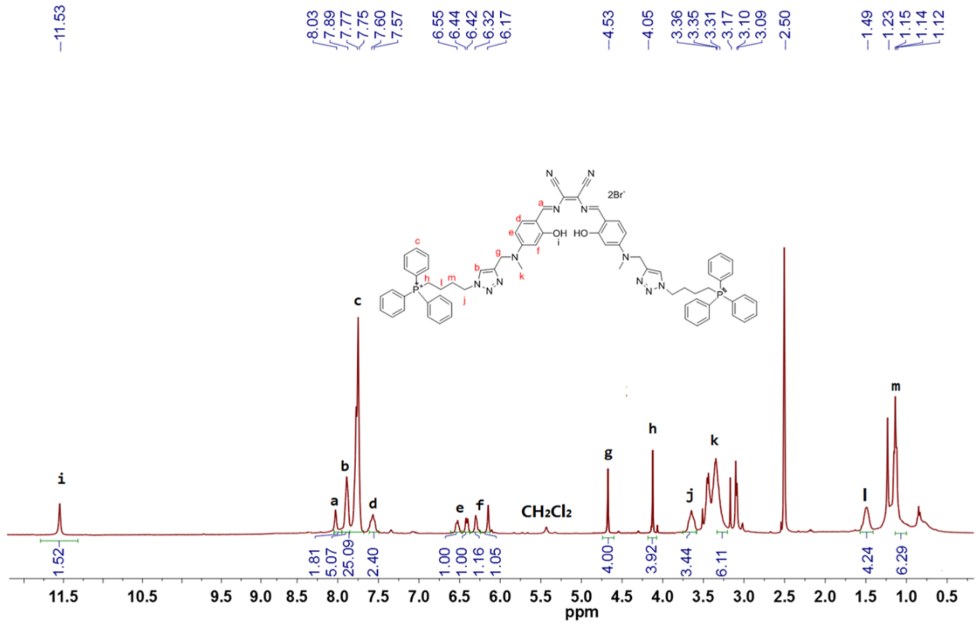

Figure S16. ^{1}H NMR spectrum of $\mathbf{L_1}$ in DMSO-d^6.

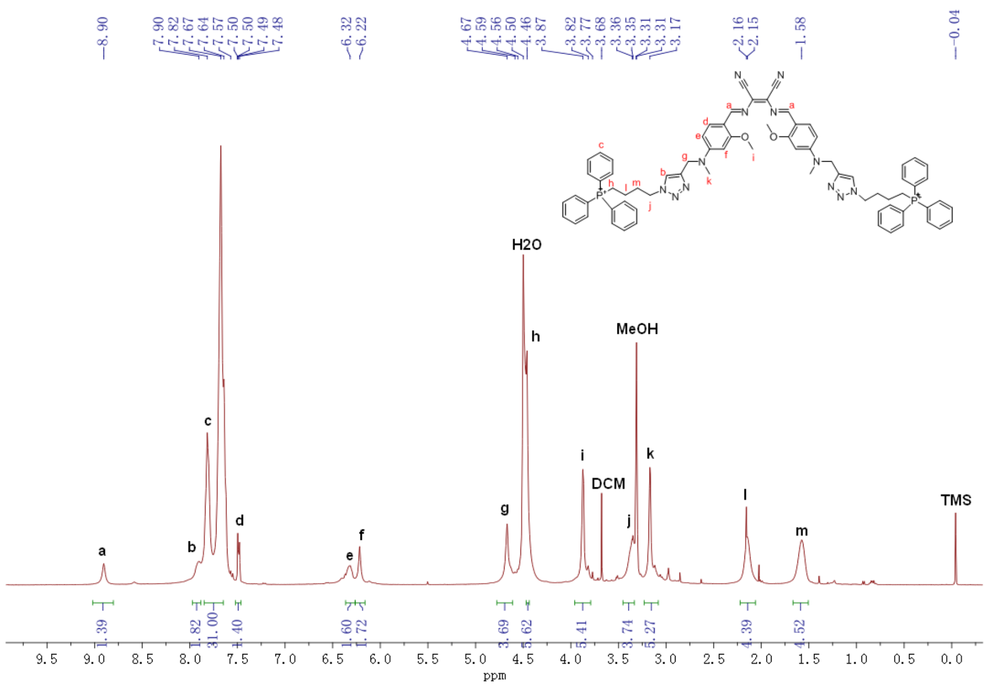

Figure S17. ^{1}H NMR spectrum of $\mathbf{L_2}$ in DMSO-d^6.

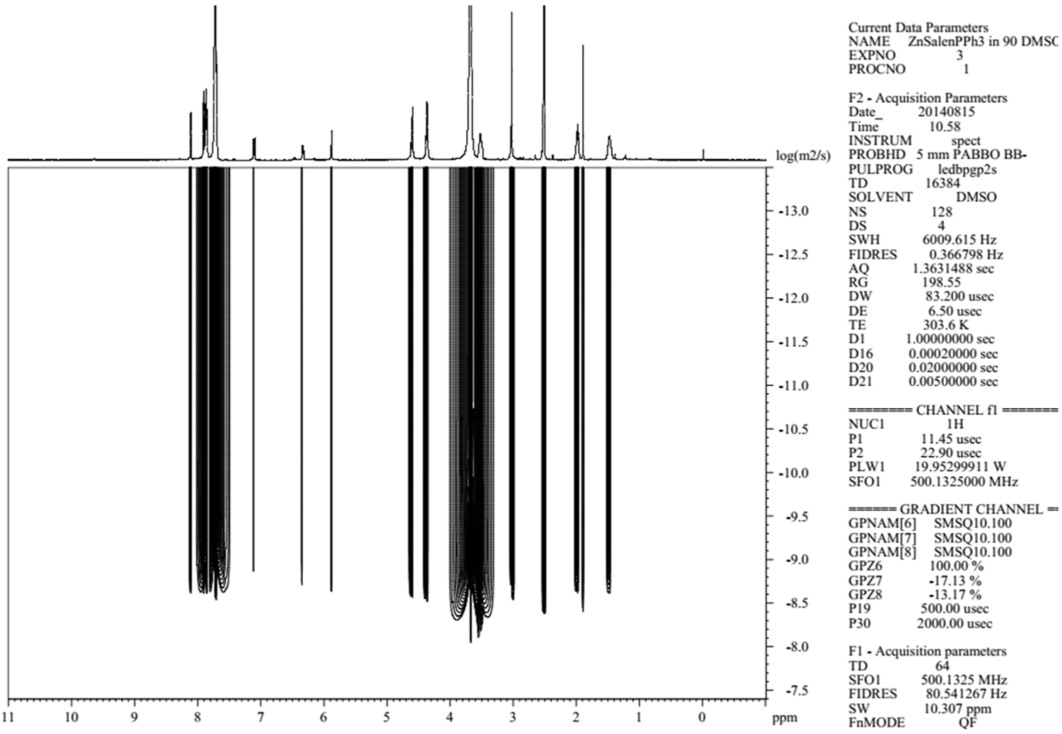

Figure S18. ^{1}H NMR DOSY spectrum of 2 mM **ZnL$_1$** in DMSO-d^6/D_2O (9:1)

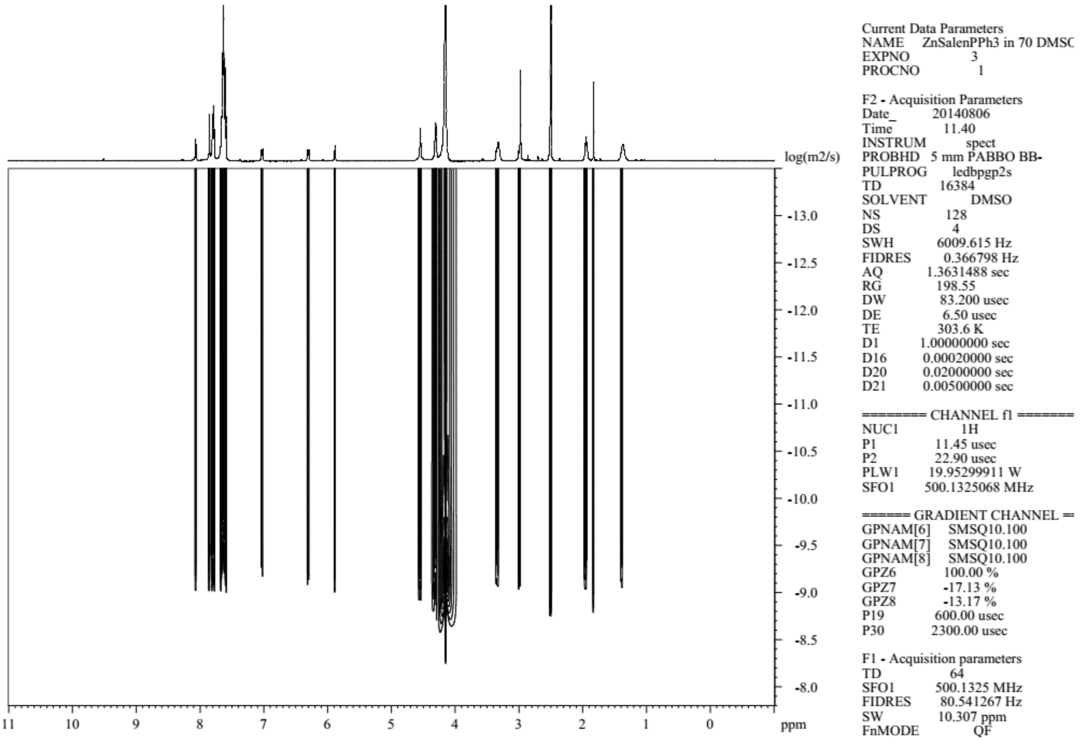

Figure S19. ^{1}H NMR DOSY spectrum of 2 mM **ZnL$_1$** in DMSO-d^6/D$_2$O (7:3)

3. IR spectra

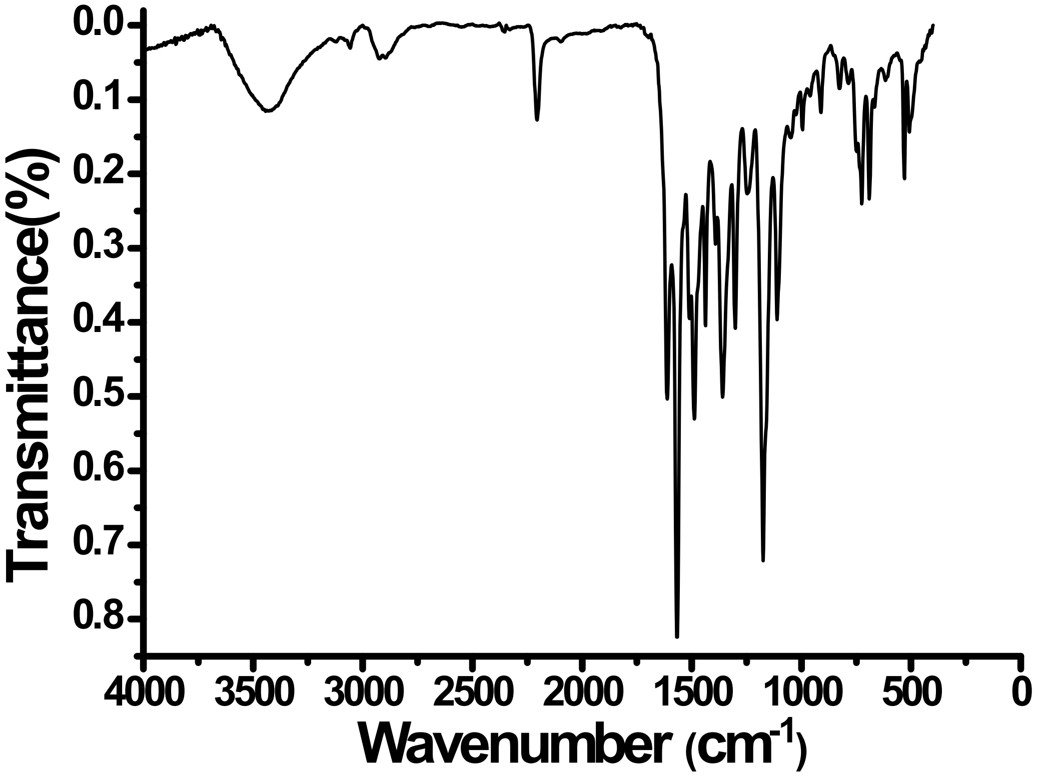

Figure S20. FT-IR spectrum (KBr pallate) of $\mathbf{ZnL_1}$.

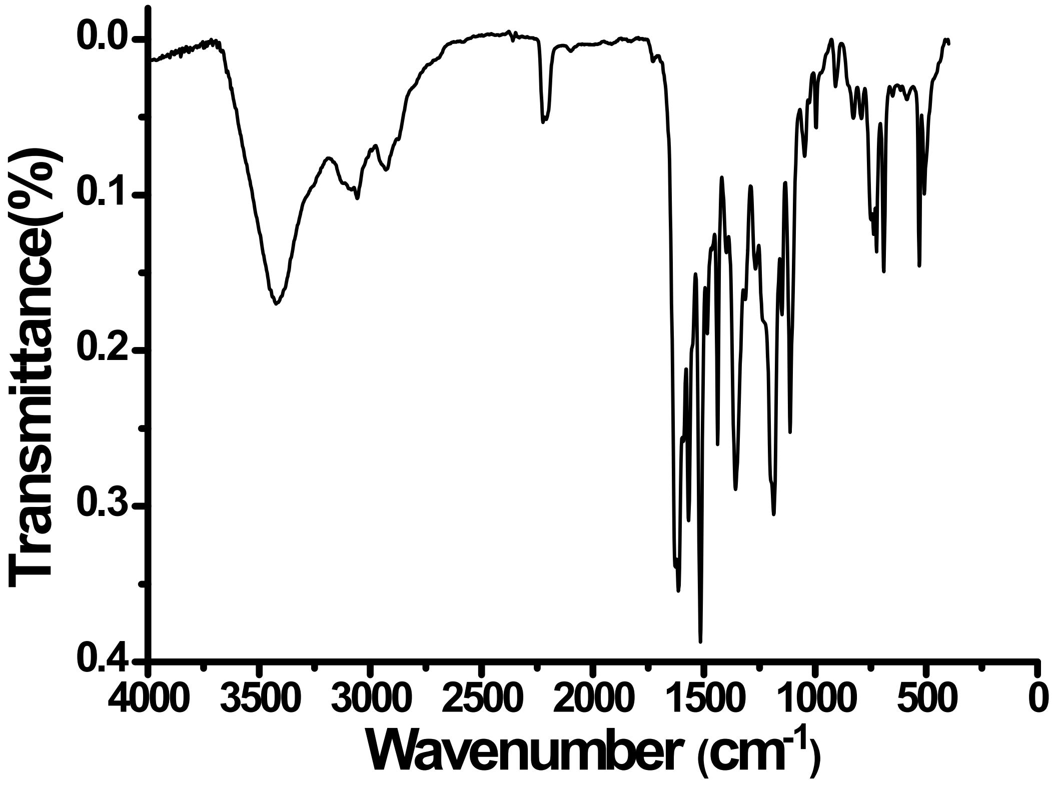

Figure S21. FT-IR spectrum (KBr pallate) of L_1.

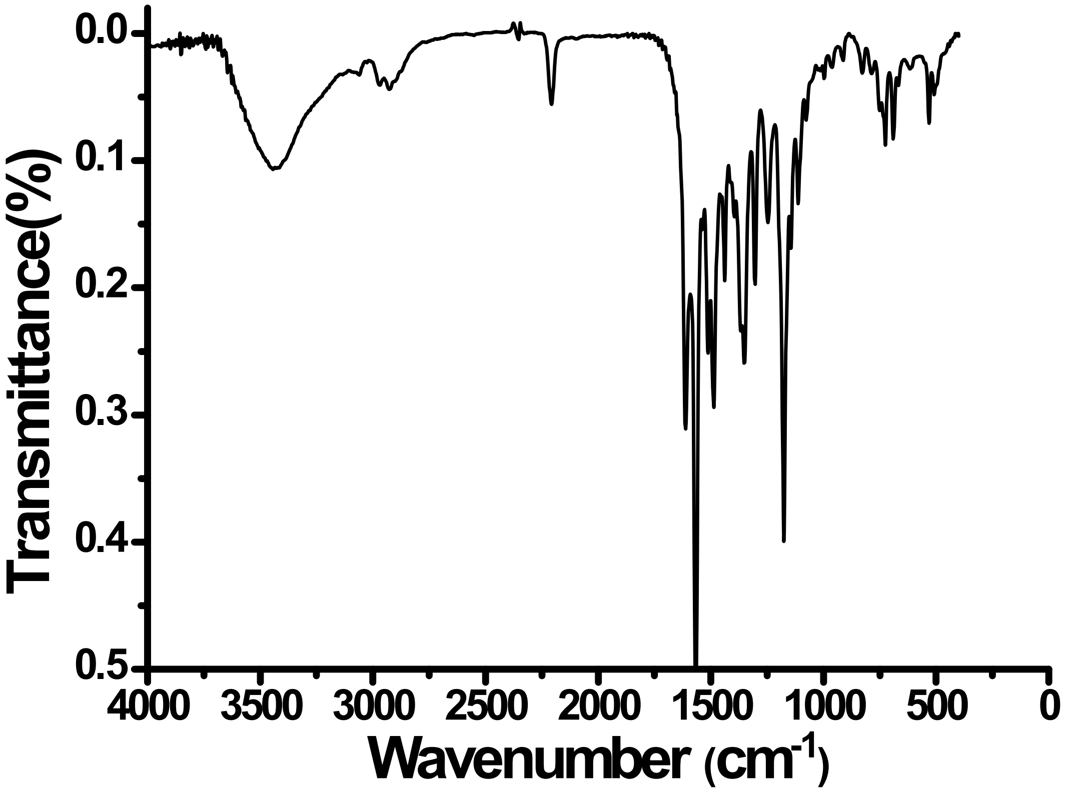

Figure S22. FT-IR spectrum (KBr pallate) of $\mathbf{L_2}$.

4.^{1}H NMR and ^{13}C NMR spectra

Compound 2 3-methoxy-N-methyl-N-(prop-2-ynyl) aniline

^{1}H NMR ($CDCl_3$, 300 M Hz)δ: 2.17 (s, 1H), 2.94 (s, 3H), 2.78 (s, 3H), 4.01 (s, 2H), 6.40 (m, 2H), 6.46 (m, 1H), 7.17 (m, 1H).

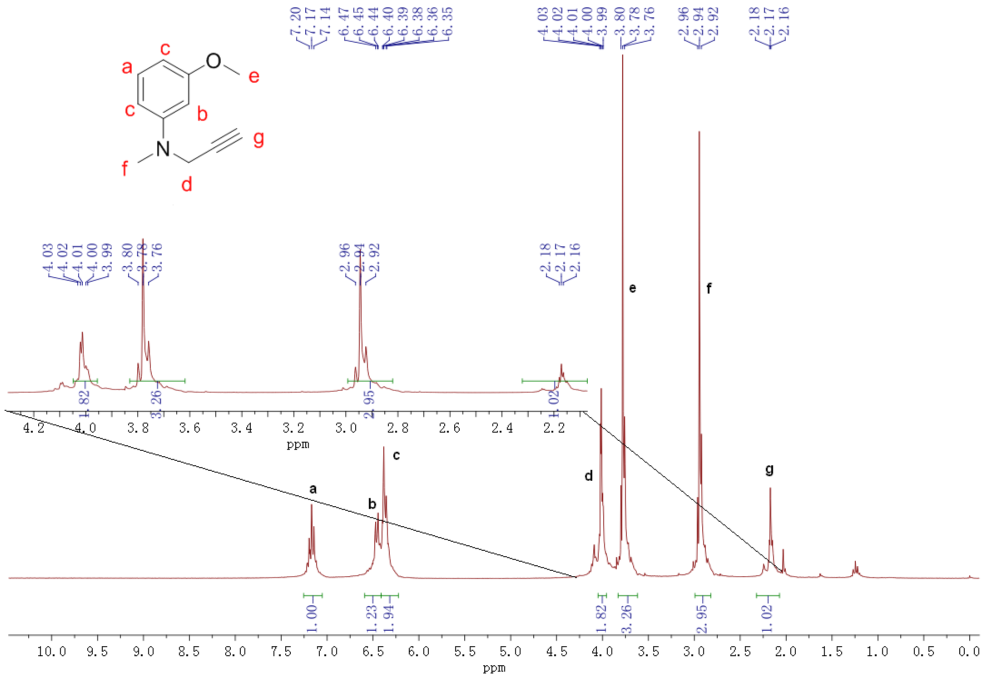

^{13}C NMR ($CDCl_3$, 100 MHz) δ: 160.71, 150.47, 129.81, 107.23, 103.13, 100.94, 79.37, 72.15, 55.16, 42.47, 38.61

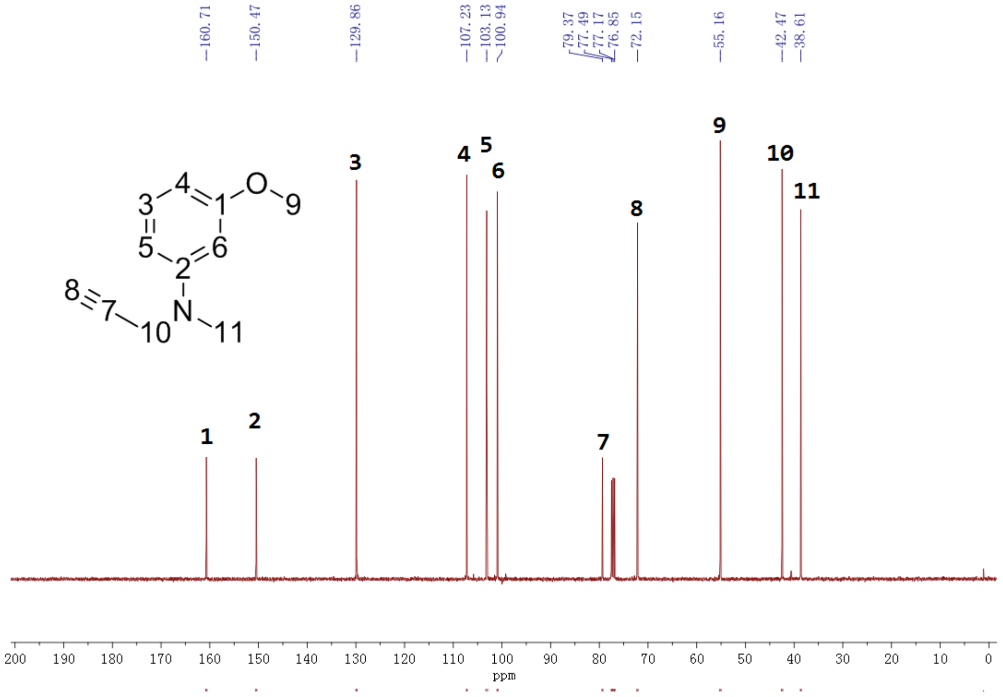

Compound 3 3-(methyl(prop-2-ynyl)amino)phenol

^{1}H NMR ($CDCl_3$, 300 M Hz): δ 2.18 (s, 1H), 2.93 (s, 3H), 4.00 (s, 2H), 5.57 (s, 1H), 6.31 (m, 2H), 6.33 (d, 1H, J = 8.4 Hz), 7.11 (t, 1H, J = 16.2 Hz).

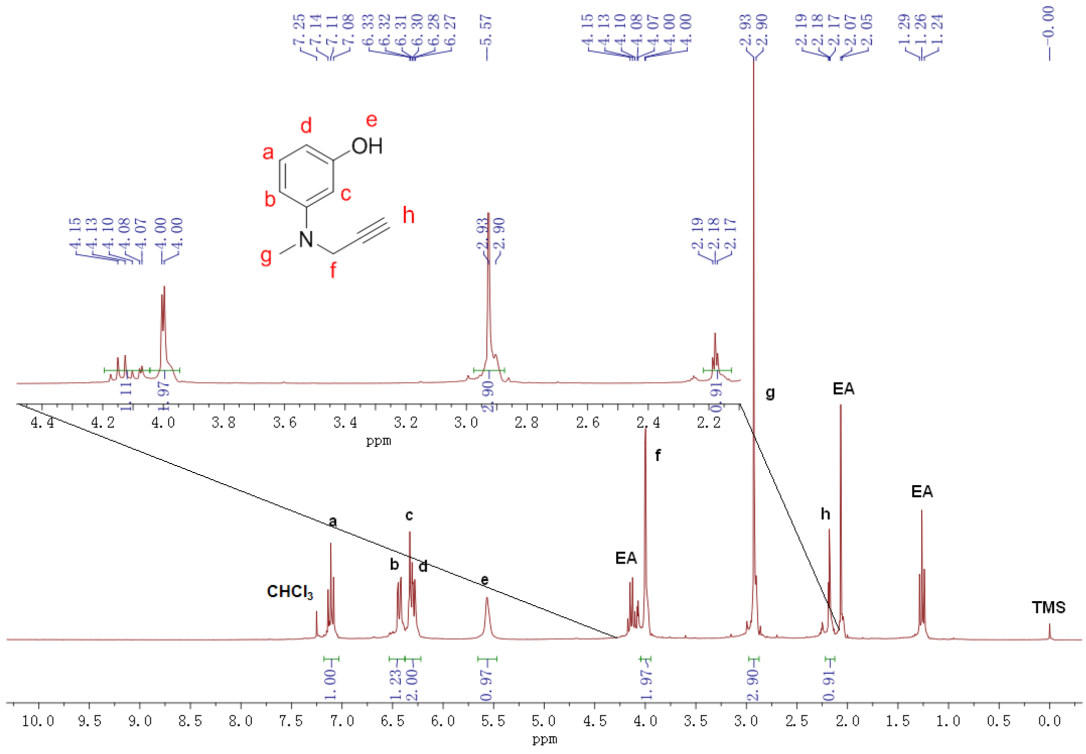

^{13}C NMR ($CDCl_3$, 100 MHz) δ: 160.94, 148.22, 130.27, 106.47, 103.29, 100.40, 80.01, 72.67, 53.39, 40.53

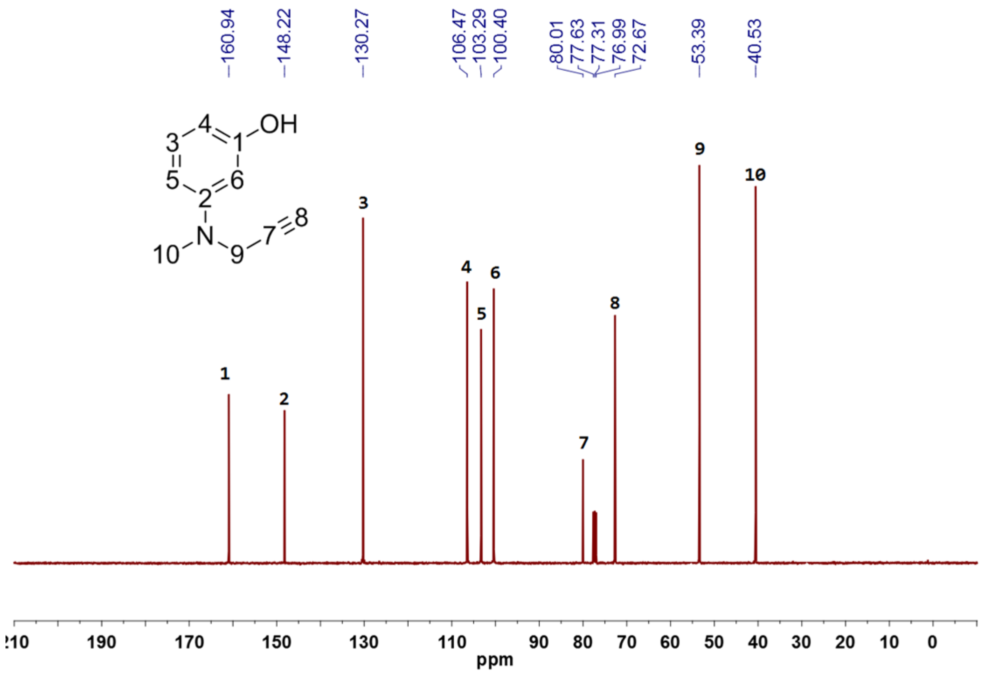

Compound 4 2-hydroxy-4-(methyl(prop-2-ynyl)amino)benzaldehyde

^{1}H NMR ($CDCl_3$, 300 M Hz): δ 2.18 (s, 1H), 3.11 (s, 3H), 4.12 (d, 2H, J = 2.4 Hz), 6.23 (d, 1H, J = 2.4 Hz), 6.40 (dd, 1H, J_1 = 8.7 Hz, J_2 = 1.8 Hz), 7.36 (d, 1H, J = 9.0 Hz), 9.59 (s, 1H), 11.55 (s, 1H).

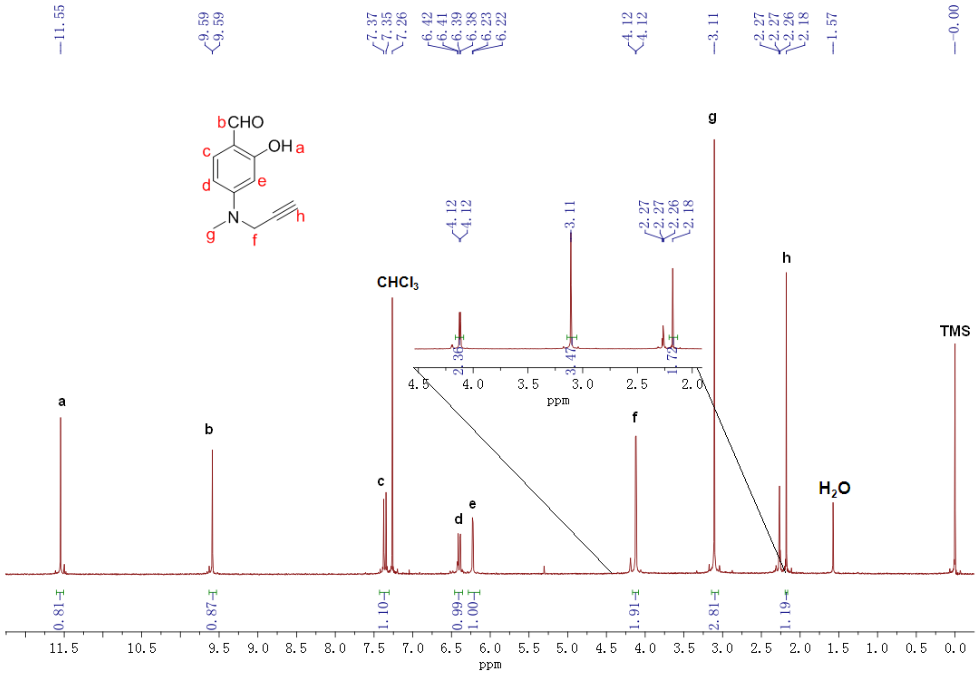

^{13}C NMR ($CDCl_3$, 100 MHz) δ: 192.94, 164.00, 155.13, 135.22, 112.64, 105.37, 98.76, 78.09, 77.39, 77.07, 76.75, 72.72, 41.74, 38.3

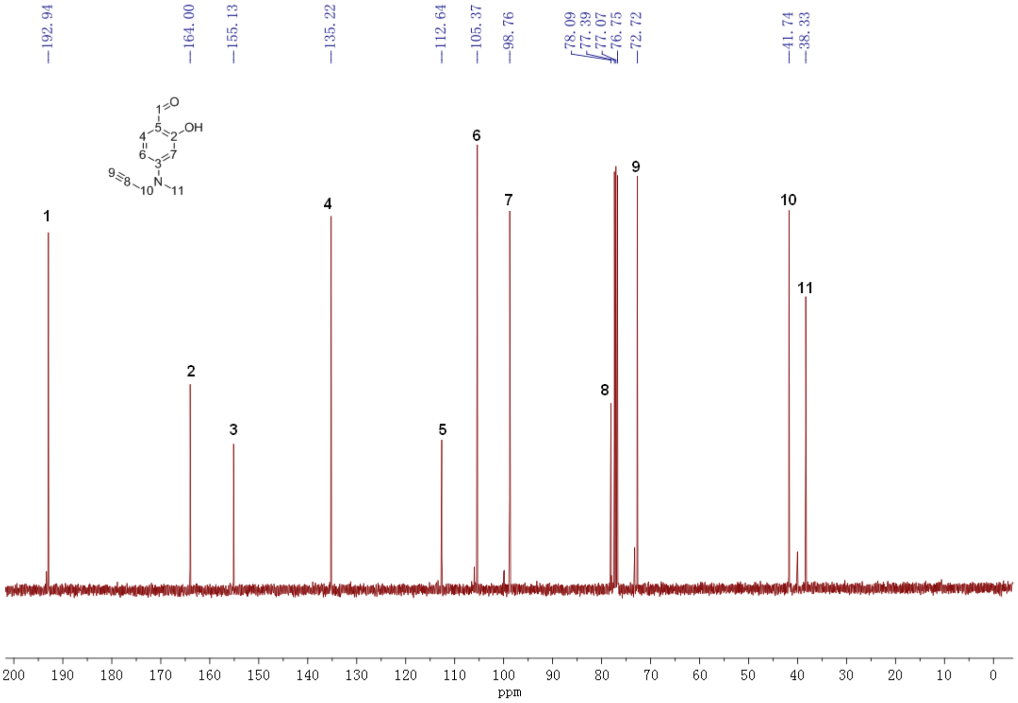

Compound 5

^{1}H NMR ($CDCl_3$, 400 MHz): δ11.43 (1H,s), 9.46 (1H,s), 8.16(1H,s), 7.77(9H, m), 7.58(6H, m), 7.24(1H, s), 6.42(1H, dd, J1=9.2 Hz, J2=2.0 Hz), 6.12(1H, d, J=2.0 Hz), 4.27(2H, s), 3.86(2H, m), 3.08(2H, s), 2.30(2H, m), 2.23(2H, s), 1.56(2H, m)

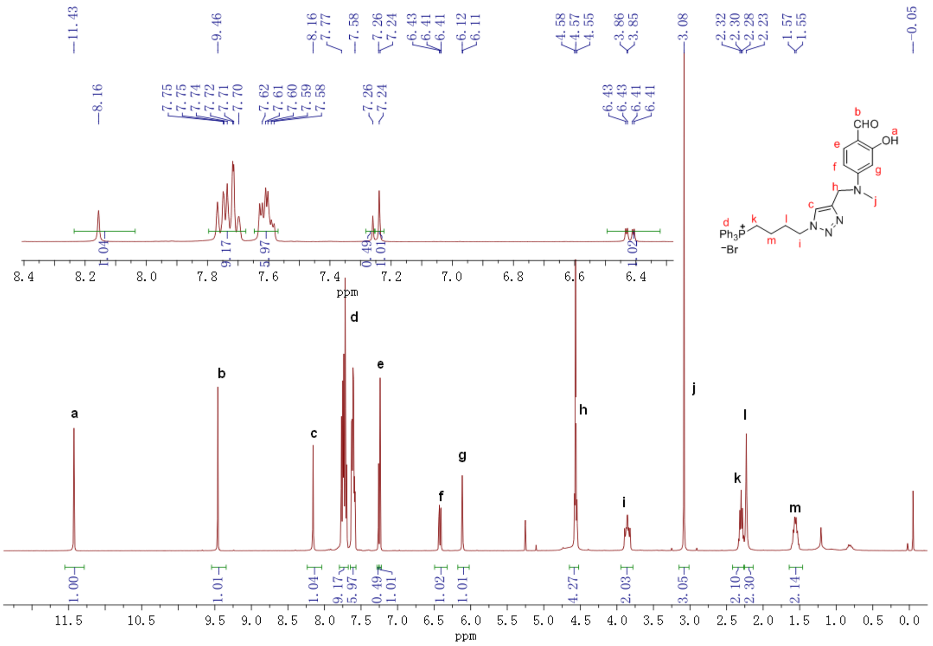

^{13}C NMR ($CDCl_3$, 100 MHz):δ 192.32, 164.04, 155.43, 143.49, 135.10, 135.05, 135.02, 133.69, 133.59, 130.52, 130.40, 123.36, 118.49, 117.63, 112.13, 105.23, 97.79,, 48.72, 47.61, 38.75, 29.96,, 21.98, 21.47.

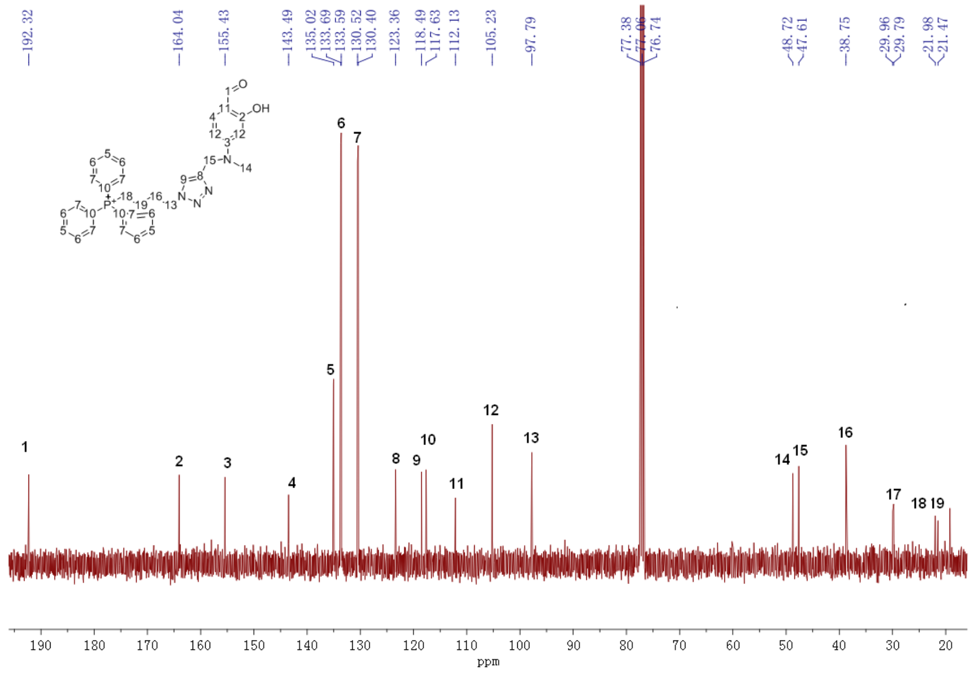

Compound 6

^{1}H NMR ($CDCl_3$, 400 MHz): δ10.14 (1H,s), 8.27 (1H,s), 7.67(9H, m), 7.63(7H, m), 6.39(1H, dd, J=9.2 Hz), 6.29(1H, s), 4.69(4H, m), 4.13(2H, m), 3.91(3H, s), 3.20(3H, s), 2.37(2H, m), 1.64(4H,

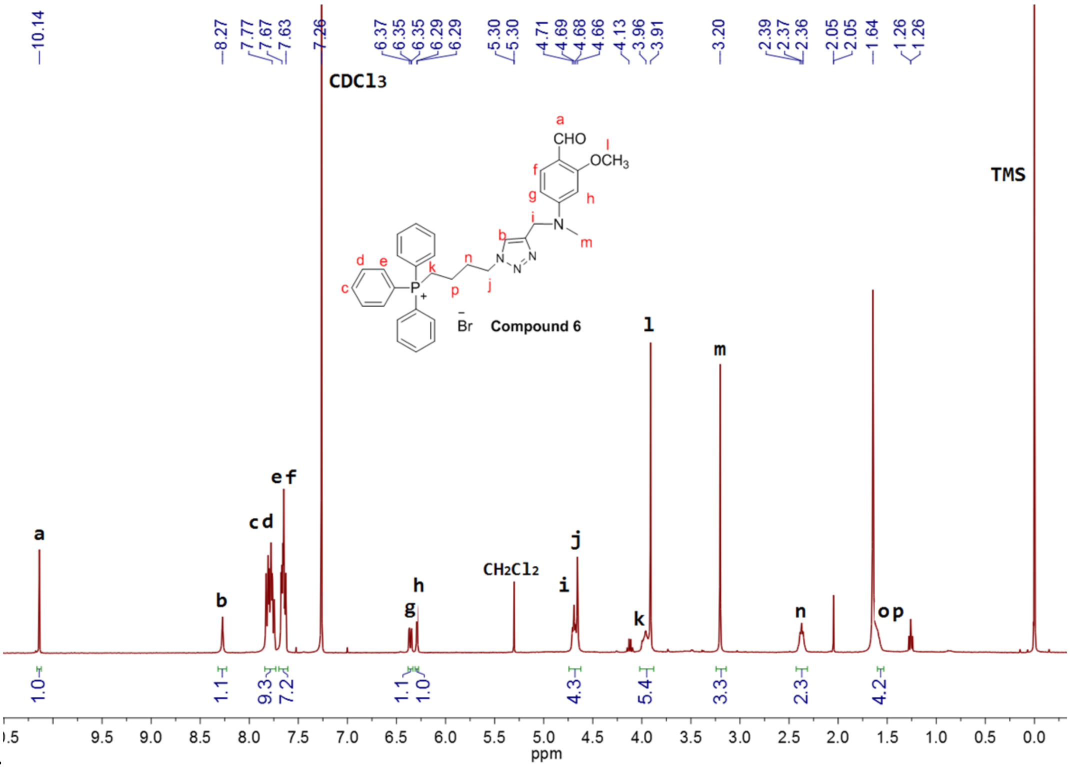

m).

^{13}C NMR ($CDCl_3$, 100 MHz), δ (ppm): 187.50, 163.86, 155.14, 135.07, 135.04, 133.71, 133.61, 130.54, 130.41, 118.47, 117.62, 114.98, 104.94, 93.94, 55.54, 48.69, 47.95, 39.06, 30.02, 29.85, 22.07, 21.56, 19.18

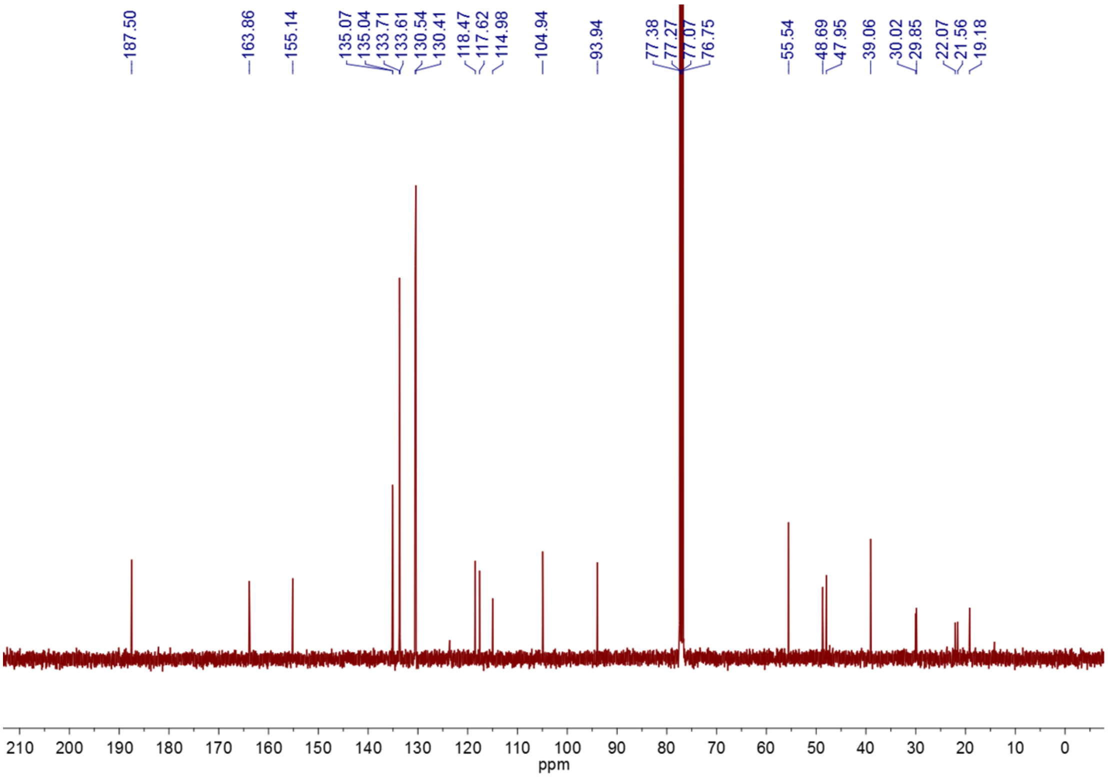

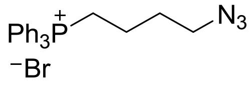

^{1}H NMR ($CDCl_3$, 400 MHz): δ7.75（15H, m）, 3.8(2H, m), 3.35(2H, m), 1.92(2H, m), 1.67(2H, m).

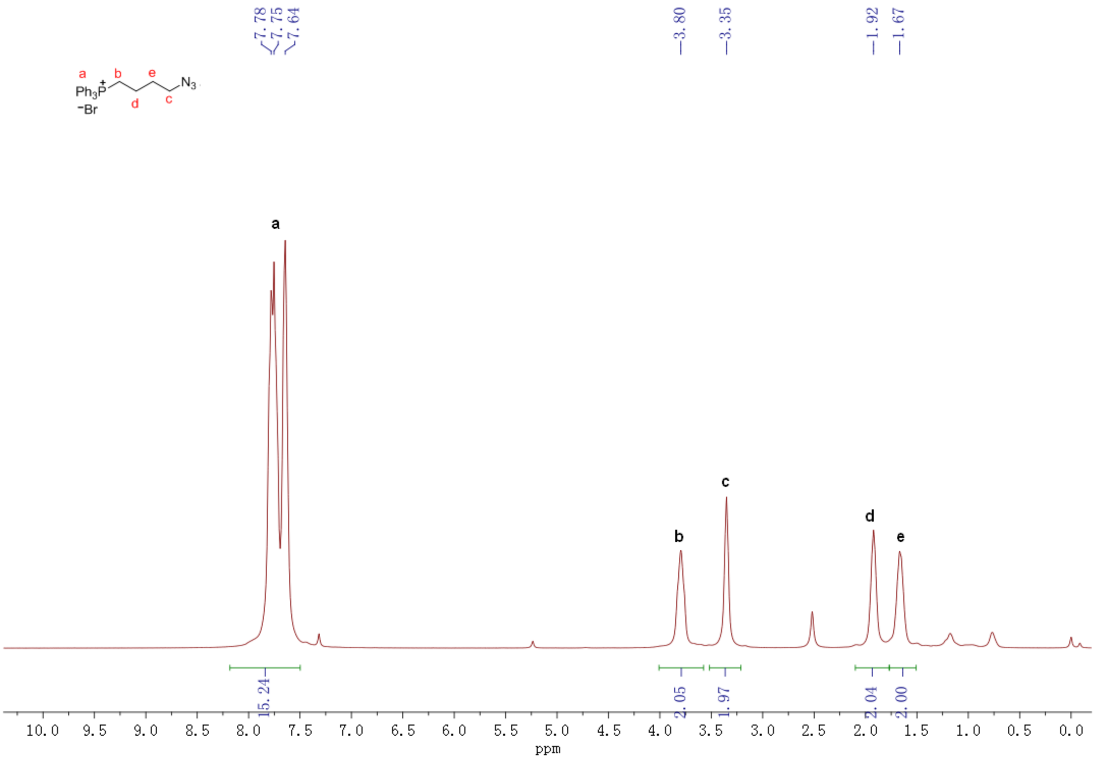

^{13}C NMR ($CDCl_3$, 100 MHz): δ 135.07, 133.52, 130.55, 118.45, 117.60, 50.50, 29.23, 22.32, 21.73

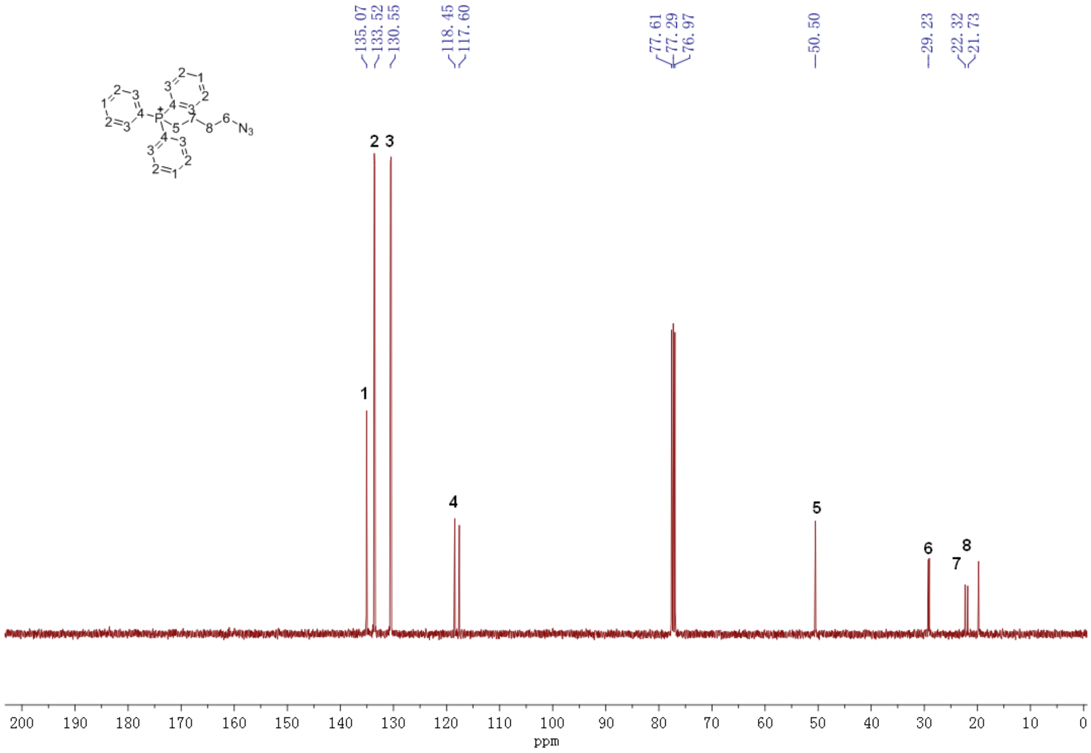

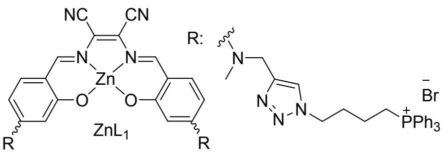

^{1}H NMR (400 MHz, d[6]DMSO): δ 8.08(s, 2H), 7.89(m, 8H), 7.75(m, 22H), 7.10(d, 2H, J=9.2 Hz), 6.30(d, 2H, J=9.2 Hz), 5.84(s, 2H), 4.61(s, 4H), 4.38(m, 4H), 3.40(m, 4H), 3.02(s, 6H), 1.96(m, 4H), 1.24(s, 4H).

The ^{1}H NMR spectrum was shown in Figure S17.

^{13}C NMR (126 MHz, DMSO): δ175.47, 155.88, 144.01, 135.46, 134.04, 133.96, 130.79, 130.69, 123.21, 119.28, 119.12, 118.43, 113.08, 102.40, 48.61, 47.14, 38.57, 30.77, 30.66, 20.38, 20.00, 19.28.

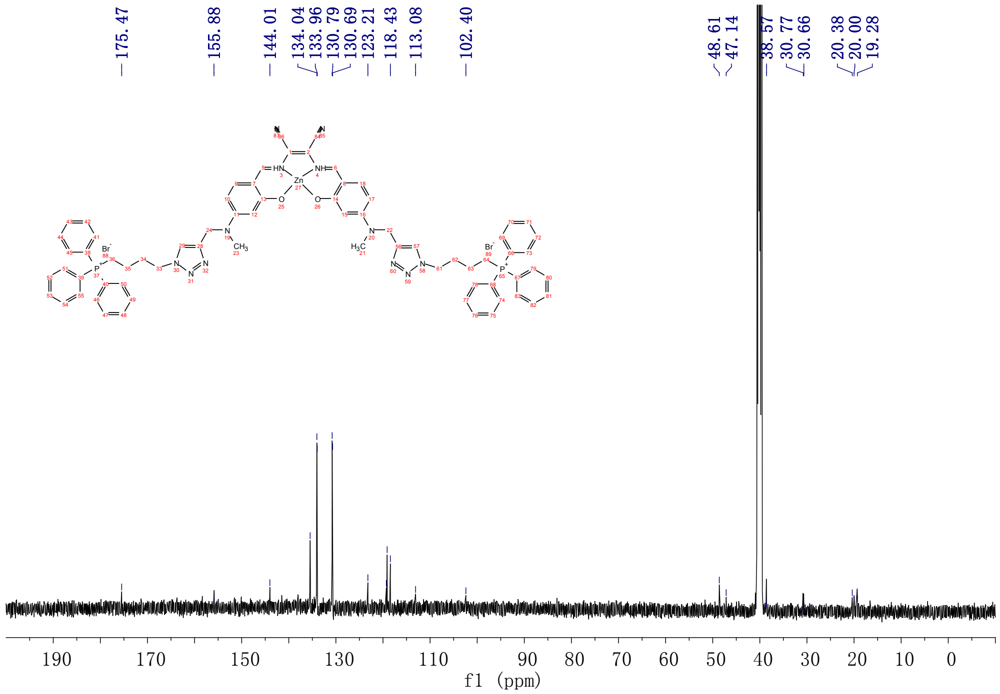

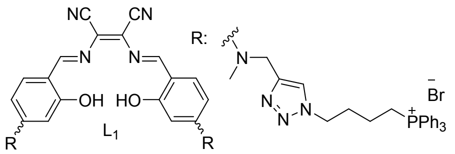

^{1}H NMR (400 MHz, d[6]DMSO): δ11.5(s, 2H), 8.03(s, 2H), 7.89-7.75(m, 30H), 7.59(m, 2H), 6.50(m, 1H), 6.42(m, 1H) 6.32(m, 1H) 6.17(m, 1H), 4.53(s, 4H), 4.05(s, 4H), 3.36(m, 4H), 3.10(s, 6H), 1.49(s, 4H), 1.14(m, 4H).

The ^{1}H NMR spectrum was shown in Figure S18.

^{13}C NMR (126 MHz, DMSO): δ163.50, 163.44, 155.50, 135.48, 134.15, 134.04, 133.96, 130.77, 130.68, 130.54, 123.32, 119.18, 118.45, 115.79, 112.46, 105.68, 97.79, 48.60, 47.19, 38.78, 31.41, 30.77, 30.68.

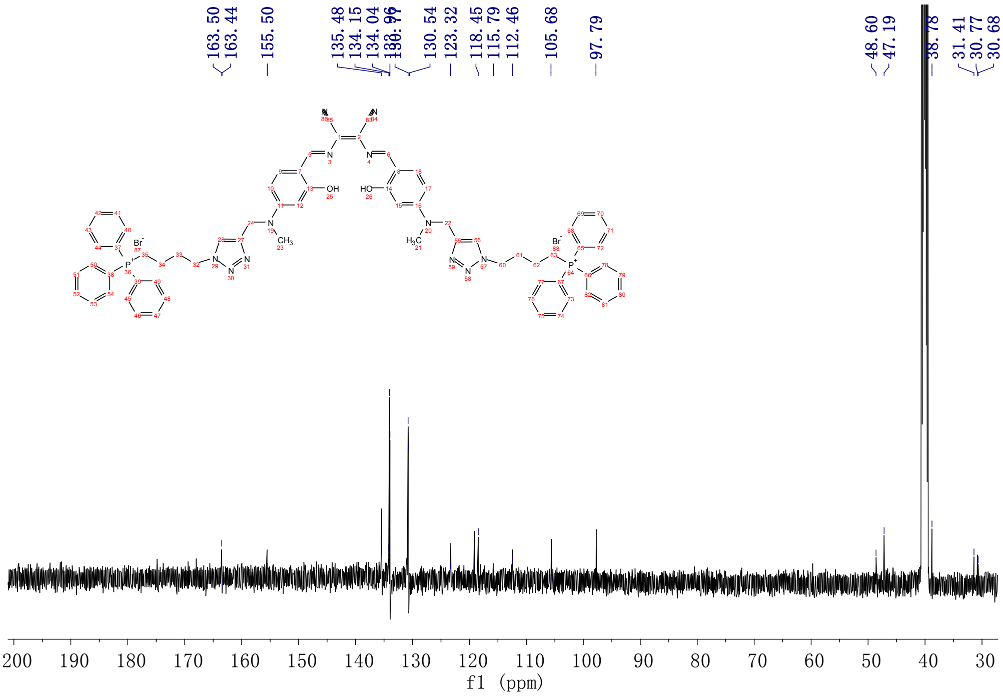

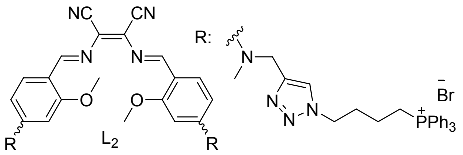

^{1}H NMR (400 MHz, MeOD): δ 8.90(s, 2H), 7.90(s, 2H), 7.82-7.50(m, 30H), 7.48(s, 2H), 6.32(s, 2H), 6.22(s, 2H), 4.67(s, 4H), 4.46(s, 4H), 3.87(s, 6H), 3.36(m, 4H), 3.17(s, 6H), 2.16(s, 4H), 1.58(s, 4H)

The ^{1}H NMR spectrum was shown in Figure S19.

^{13}C NMR (126 MHz, DMSO): δ135.42, 134.03, 133.95, 130.78, 130.68, 55.36.

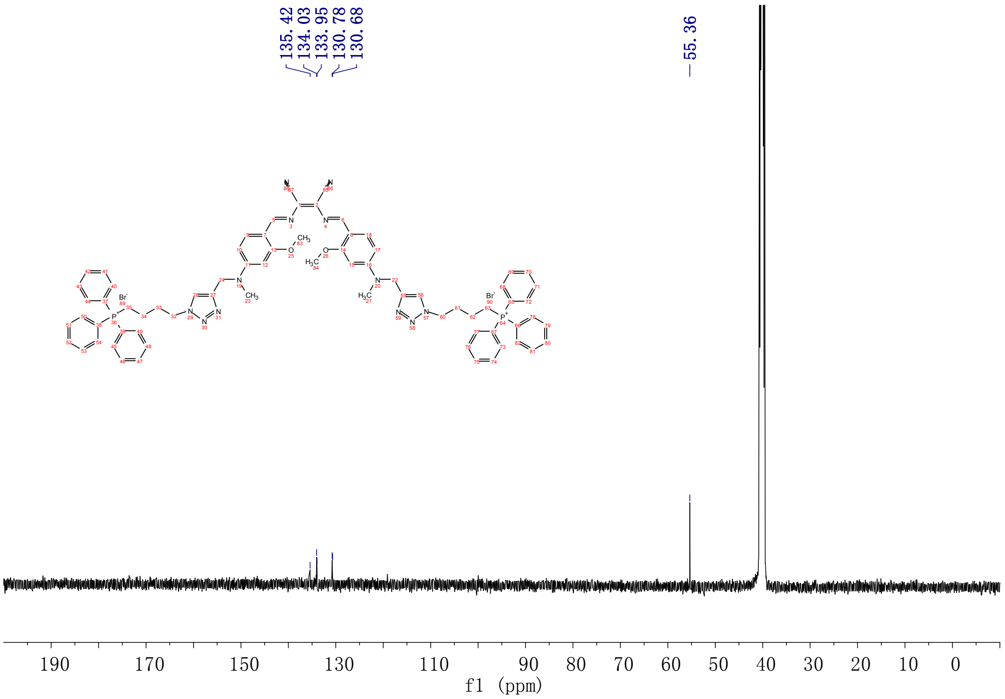

5. HR ESI-MS spectra

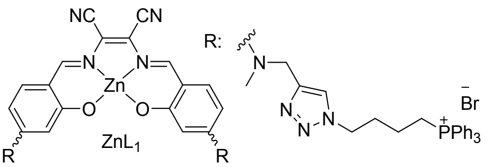

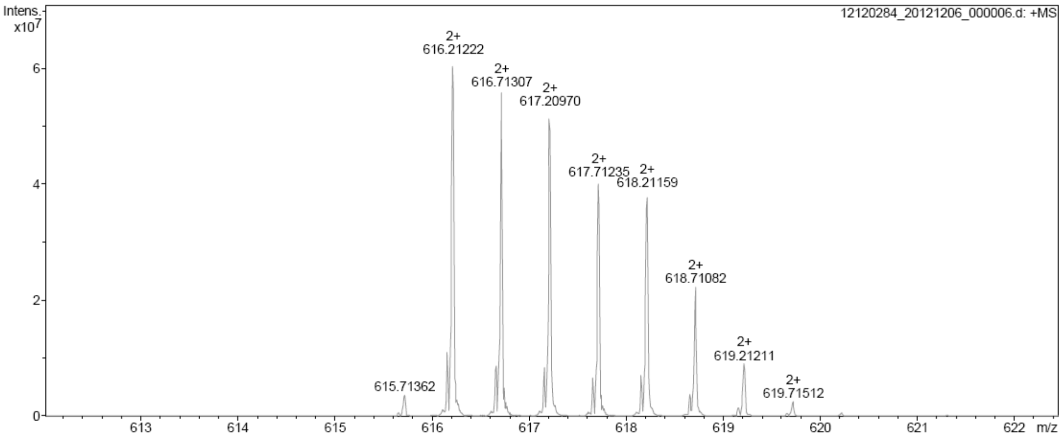

Meas. m/z	#	Formula	Score	m/z	err [mDa]	err [ppm]	mSigma	rdb	e⁻ Conf	N-Rule
616.21222	1	C 70 H 66 N 12 O 2 P 2 Zn	100.00	616.20937	-2.9	-4.6	40.3	45.0	even	ok

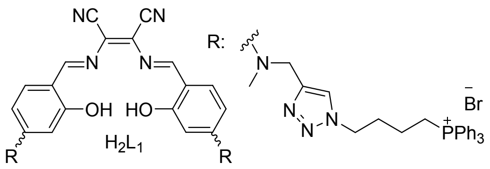

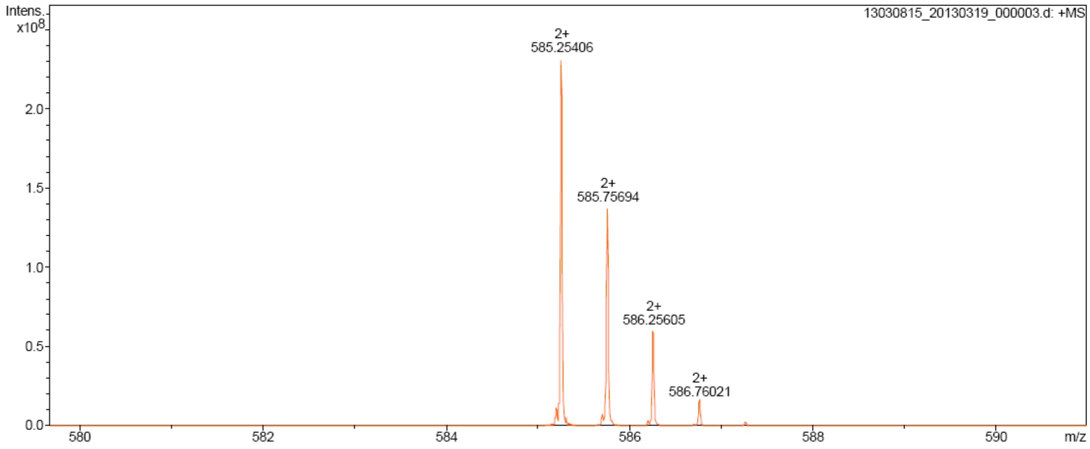

Meas. m/z	#	Formula	Score	m/z	err [mDa]	err [ppm]	mSigma	rdb	e^- Conf	N-Rule
585.25406	1	C 70 H 68 N 12 O 2 P 2	100.00	585.25262	-1.4	-2.5	98.9	44.0	even	ok

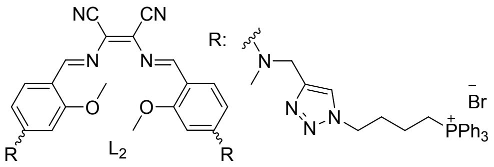

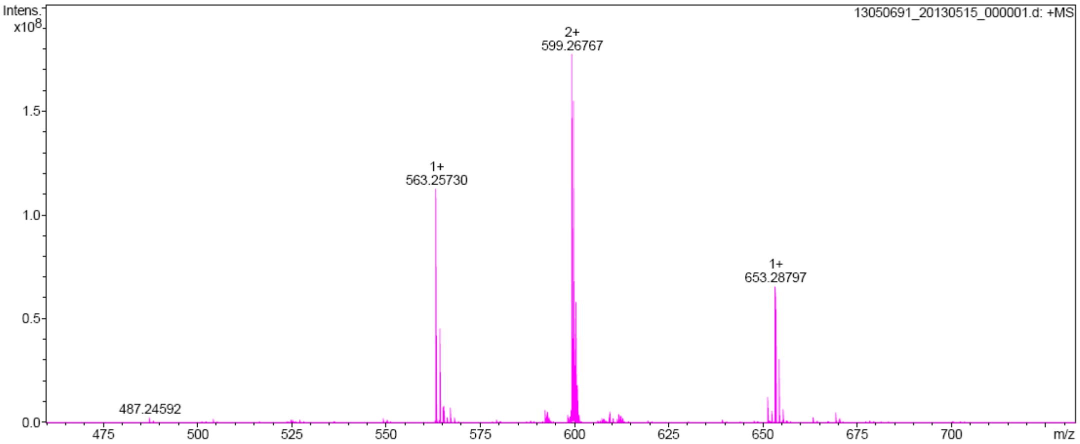

Meas. m/z	#	Formula	Score	m/z	err [mDa]	err [ppm]	mSigma	rdb	e^{-} Conf	N-Rule
599.26767	1	C 72 H 72 N 12 O 2 P 2	100.00	599.26827	0.6	1.0	34.2	44.0	even	ok

6. References

[1] P. G. Lacroix, S. Di Bella, I. Ledoux, *Chem. Mater.* **1996**, *8*, 541-545.

[2] R. F. Kubin, A. N. Fletcher, *J. Lumin.* **1983**, *27*, 455-462.

[3] T. Karstens, K. Kobs, *J. Phys. Chem.* **1980**, *84*, 1871-1872.

[4] C. Xu, W. W. Webb, *J. Opt. Soc. Am. B* **1996**, *13*, 481-491.

[5] E. J. New, A. Congreve, D. Parker, *Chem Sci* **2010**, *1*, 111-118.

[6] I. G. Zigoneanu, Y. J. Yang, A. S. Krois, M. E. Haque, G. J. Pielak, *BBA-Biomembranes* **2012**, *1818*, 512-519.

[7] G. Salassa, M. J. J. Coenen, S. J. Wezenberg, B. L. M. Hendriksen, S. Speller, J. A. A. W. Elemans, A. W. Kleij, *J. Am. Chem. Soc.* **2012**, *134*, 7186-7192.

[8] G. Consiglio, S. Failla, P. Finocchiaro, I. P. Oliveri, R. Purrello, S. Di Bella, *Inorg. Chem.* **2010**, *49*, 5134-5142.
